# Structure and function analysis of a type III preQ_1_-I riboswitch from *Escherichia coli* reveals direct metabolite sensing by the Shine-Dalgarno sequence

**DOI:** 10.1016/j.jbc.2023.105208

**Published:** 2023-09-01

**Authors:** Griffin M. Schroeder, Daniil Kiliushik, Jermaine L. Jenkins, Joseph E. Wedekind

**Affiliations:** 1 Department of Biochemistry and Biophysics, University of Rochester School of Medicine and Dentistry, Rochester, New York, USA; 2 Center for RNA Biology, University of Rochester School of Medicine and Dentistry, Rochester, New York, USA

**Keywords:** gene regulation, preQ_1_-I riboswitch, x-ray crystallography, Shine-Dalgarno sequence, pseudoknot, molecular recognition, translation regulation

## Abstract

Riboswitches are small noncoding RNAs found primarily in the 5′ leader regions of bacterial messenger RNAs where they regulate expression of downstream genes in response to binding one or more cellular metabolites. Such noncoding RNAs are often regulated at the translation level, which is thought to be mediated by the accessibility of the Shine-Dalgarno sequence (SDS) ribosome-binding site. Three classes (I-III) of prequeuosine_1_ (preQ_1_)-sensing riboswitches are known that control translation. Class I is divided into three subtypes (types I-III) that have diverse mechanisms of sensing preQ_1_, which is involved in queuosine biosynthesis. To provide insight into translation control, we determined a 2.30 Å-resolution cocrystal structure of a class I type III preQ_1_-sensing riboswitch identified in *Escherichia coli* (*Eco*) by bioinformatic searches. The *Eco* riboswitch structure differs from previous preQ_1_ riboswitch structures because it has the smallest naturally occurring aptamer and the SDS directly contacts the preQ_1_ metabolite. We validated structural observations using surface plasmon resonance and *in vivo* gene-expression assays, which showed strong switching in live *E. coli*. Our results demonstrate that the *Eco* riboswitch is relatively sensitive to mutations that disrupt noncanonical interactions that form the pseudoknot. In contrast to type II preQ_1_ riboswitches, a kinetic analysis showed that the type III *Eco* riboswitch strongly prefers preQ_1_ over the chemically similar metabolic precursor preQ_0_. Our results reveal the importance of noncanonical interactions in riboswitch-driven gene regulation and the versatility of the class I preQ_1_ riboswitch pseudoknot as a metabolite-sensing platform that supports SDS sequestration.

Riboswitches are small noncoding RNAs found primarily in the 5′-leader regions of bacterial messenger RNAs (1, 2). These noncoding RNAs regulate expression of downstream genes in response to binding one or more cellular metabolites or ions in the aptamer domain that in turn exposes or sequesters regulatory sequences in a downstream expression platform (1, 2, 3, 4). Riboswitch-driven gene regulation typically occurs at the level of translation initiation or transcription termination (5, 6). Importantly, riboswitches participate in biochemical feedback loops by selectively sensing a metabolite that is related to a downstream gene (1, 2).

Of the over 55 validated riboswitch classes (1), the prequeuosine_1_ (preQ_1_)-sensing riboswitch family was one of the first to be identified (7) and validated (8). The preQ_1_ family can be divided into three classes. All three sense preQ_1_ (9), a 7-aminomethyl-7-deazaguanine metabolite that is the last metabolic precursor in the production of the hypermodified nucleoside queuosine (10, 11) (Fig. 1*A*). Lack of queuosine can diminish bacterial virulence (12), making preQ_1_ riboswitches candidates for drug targeting.

**Figure 1:**
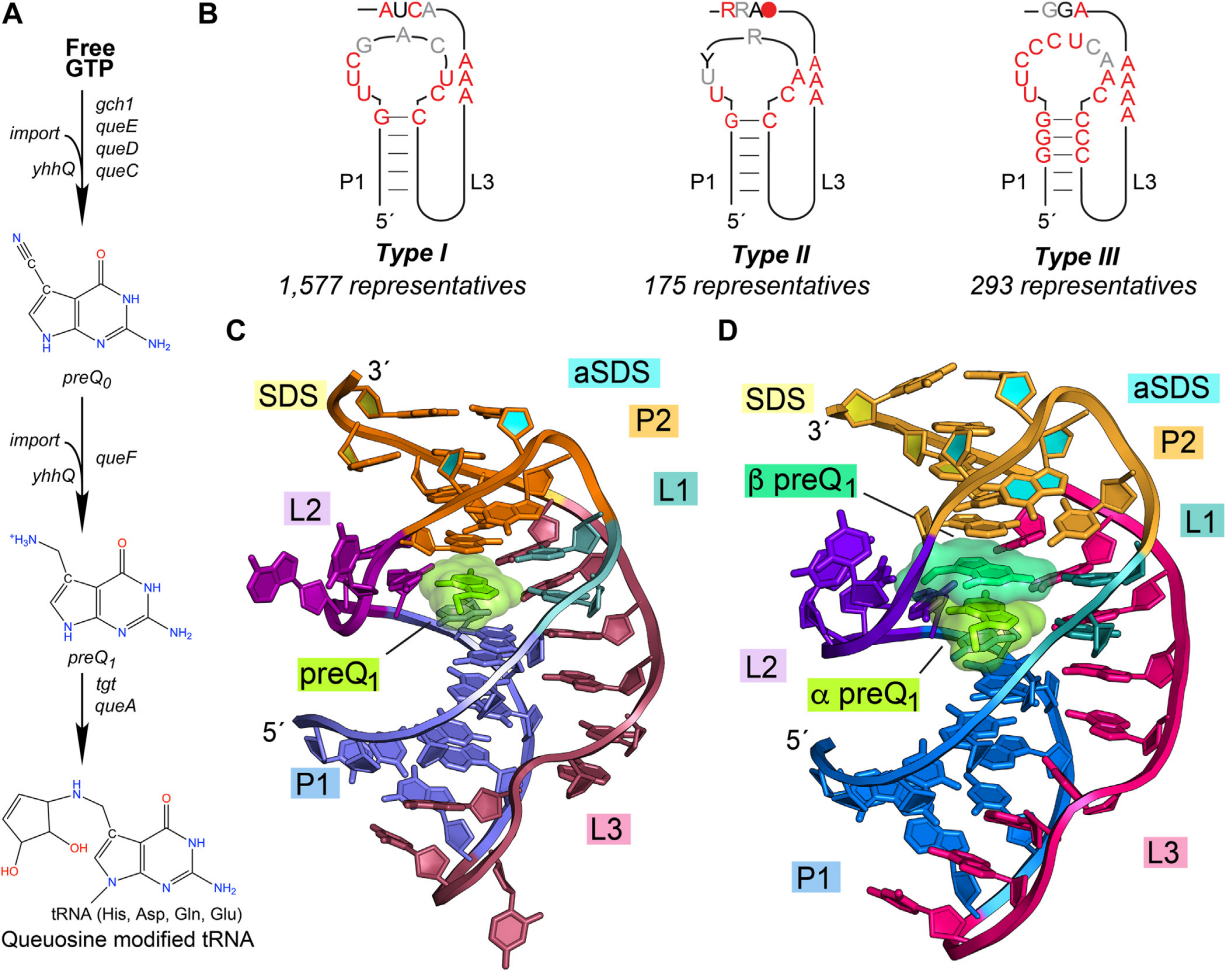
**PreQ**_**1**_**biosynthesis, consensus models of class I preQ**_**1**_**riboswitches, and representative type I and II cocrystal structures.***A*, prokaryotic *de novo* queuosine biosynthetic pathway. Free GTP is first enzymatically converted to preQ_0_ by *gch1, queE, queD*, and *queC* gene products and is then converted to preQ_1_ by *queF* (10, 65). The *yhhQ* transporter can salvage both preQ_0_ and preQ_1_ from the environment in proteobacteria (28). *Tgt*-derived enzymes insert preQ_1_ at position 34 of tRNAs containing GUN anticodons whereupon *queA* modifies preQ_1_ into queuosine (10, 11). *B*, covariation diagrams of each preQ_1_ riboswitch subtype with nucleotides in *red*, *black*, and *gray* indicating 97%, 90%, and 75% sequence conservation (9). Ribbon diagrams of cocrystal structures for the (*C*) type II *Tte* (PDB code 6vui) (21) and (*D*) type I *Can* (PDB code 8fb3) riboswitches (47). Positions are colored according to pseudoknot pairing and loop regions. PreQ_1_ metabolites are depicted as *green* surface models. preQ1, prequeuosine1.

The class I preQ_1_ (preQ_1_-I) riboswitch is the smallest known naturally occurring ligand-binding aptamer and the most abundant of the three preQ_1_ classes as indicated by its broad distribution across numerous bacterial phyla (8, 9). Class I can be divided further into three subtypes, type I-III (9) (Fig. 1*B*). High-resolution crystal structures of class I type II preQ_1_-I (preQ_1_-I_II_) riboswitches from *Thermoanaerobacter tengcongenesis* (*Tte*) (13, 14) (Fig. 1*C*) and *Bacillus subtilis* (15) revealed a compact H-type pseudoknot fold that recognizes a single preQ_1_ molecule. These crystal structures spurred numerous single-molecule investigations (16, 17, 18, 19), computational studies (20) and biophysical analyses (21) that probed folding pathways (17, 19), gene-regulatory mechanisms (16, 18), and conformational interconversion pathways (20, 21). In addition, class I type II preQ_1_-I_II_ riboswitches inspired design of drug-like small molecules that modulate biological function (22, 23).

Multiple efforts have continued to identify new riboswitch classes (24), although riboswitch variants within the same class can exhibit astonishing diversity in terms of metabolite recognition, despite similar tertiary structure and homologous sequence (25). For example, we recently discovered that class I type I preQ_1_ (*i.e.*, preQ_1_-I_I_) riboswitches sense two equivalents of preQ_1_ through interactions in which metabolites hydrogen bond and π-stack with one other in the same binding pocket (26) (Fig. 1*D*). This work demonstrated how structural studies on different sequence cohorts within the same riboswitch class can reveal new modes of RNA-small molecule recognition, providing insight into the chemical diversity of RNA, and its potential for ligand recognition and catalysis in an RNA world (26).

The class I type III preQ_1_ (*i.e.*, preQ_1_-I_III_) riboswitch subclass was identified in a bioinformatic search carried out after the type I and II subgroups were identified (9). Currently there is no high-resolution structure for any preQ_1_-I_III_ riboswitch. The type III covariation model predicted the presence of an H-type pseudoknot fold similar to types I and II (Fig. 1*B*). However, the type III riboswitch has a shorter pyrimidine-rich hairpin loop and a longer 3′-tail having a variable length that comprises A-rich sequences that link the P1 helix to the expression platform (Fig. 1*B*). The ability of the preQ_1_-I_III_ riboswitch to bind preQ_1_ was validated previously using in-line probing with representative sequences derived from *Shigella dysenteriae* (9) and isothermal titration calorimetry (ITC) with sequences from *Enterobacter cloacae* (27). Each technique suggested that class I type III riboswitches recognize one ligand equivalent. Interestingly, the type III preQ_1_ riboswitch subgroup is found almost exclusively in γ−proteobacteria where they regulate expression of the preQ_0_/preQ_1_ scavenging gene *yhhQ* (28). This observation suggested that—like other class I subtypes—type III riboswitches might not discriminate between preQ_1_ and preQ_0_ (Fig. 1*A*) (13).

To investigate the mode of metabolite recognition by preQ_1_-I_III_ riboswitches and Shine-Dalgarno sequence (SDS) sequestration that represses gene expression, we determined the cocrystal structure of a preQ_1_-I_III_ riboswitch from *Escherichia coli*, termed *Eco*, to 2.30 Å resolution. Phasing by single-wavelength anomalous diffraction (SAD) used Mn^2+^ ions that mimic naturally occurring divalent ion binding sites. The structure revealed an H-type pseudoknot fold that recognizes preQ_1_. The type III *Eco* riboswitch sequesters its SDS through predicted (9) pseudoknot interactions that involve the novel pyrimidine-rich aptamer loop. Unexpectedly, the SDS also directly senses preQ_1_, which is in contrast to the mechanisms used by all other known preQ_1_ riboswitches (13, 14, 22, 29, 30, 31). To validate the mode of preQ_1_ recognition, we used surface plasmon resonance (SPR) and a reporter-gene assay in live *E. coli* to detect riboswitch function. Mutations that disrupt the noncanonical interactions in the binding pocket were tolerated poorly compared to other class I riboswitches. Our results also revealed a high level of kinetic discrimination for preQ_0_ relative to preQ_1_. Overall, our findings detail an economical aptamer that directly utilizes its expression platform for metabolite sensing, underscoring the diverse strategies by which riboswitches control translation.

## Results

### Type III riboswitch identification and decreased L3 linker size to improve preQ_1_ affinity

We recently discovered a novel mode of preQ_1_ recognition for class I type I (preQ_1_-I_I_) riboswitches that went undiscovered for 15 years (26). To characterize ligand binding and gene regulation by preQ_1_-I_III_ riboswitches, we searched for sequences having small aptamers adjacent to strong SDSs that would be amenable to switching in our *E. coli*–based reporter coupled assay (32). A BLAST search on the NCBI server using the published covariation model identified a 36-mer *Eco* riboswitch sequence (9). This *Eco* preQ_1_-I_III_ riboswitch was associated with a downstream *yhhQ* gene that recently was demonstrated to encode a preQ_0_ or preQ_1_ transporter (28).

**Figure 2:**
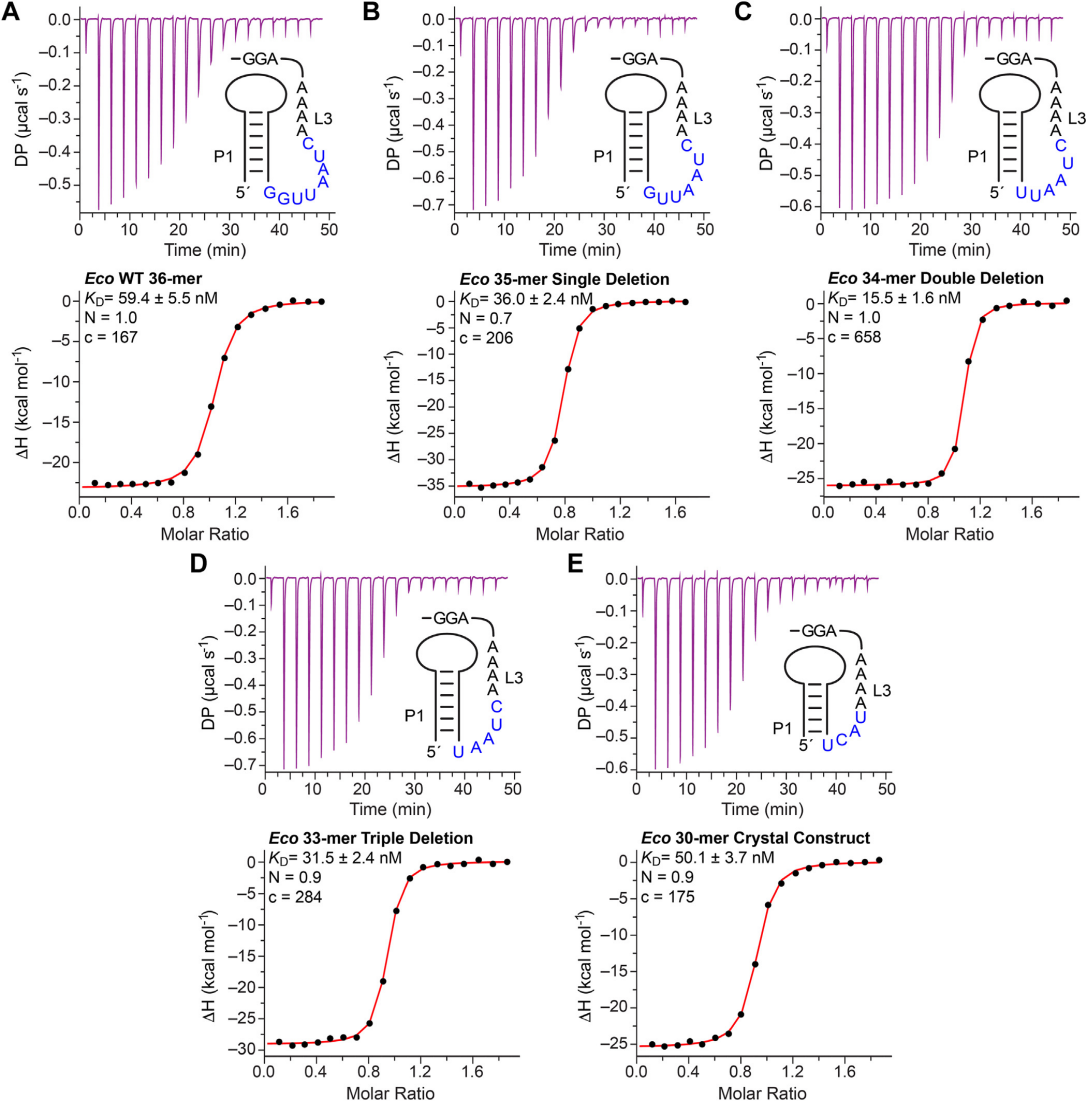
**Length dependence of loop L3 on metabolite binding to the class I type III preQ**_**1**_**(preQ**_**1**_**-I**_**III**_**) riboswitch from *E. coli*.***A*, representative isothermal titration calorimetry (ITC) thermogram for preQ_1_ binding to the 36-mer WT *Eco*. *Inset:* schematic diagram of the L3 loop sequence that was varied in this analysis. *K*_D_, N (stoichiometry), and *C* values are shown. *B*, single-deletion ΔG *Eco* 35-mer variant. *C*, double deletion ΔGG *Eco* 34-mer variant. *D*, triple deletion ΔGGU *Eco* 33-mer variant. *E*, *Eco* 30-mer crystal construct using a linker based on the *Tte* riboswitch P1-L3 transition. All experiments were performed in duplicate and average thermodynamic parameters are provided in Table 1.

We next used ITC to characterize ligand binding by the *Eco* riboswitch sequence. Since preQ_0_ has comparatively limited solubility in solution, we tested preQ_1_ binding (13). The WT riboswitch had an average *K*_D_ of 57.9 ± 1.5 nM in a reaction driven by favorable enthalpy (Δ*H* of −23.1 ± 0.3 kcal mol^−1^) that offsets an unfavorable entropy (–TΔ*S* of +13.2 ± 0.3 kcal mol^−1^) (Table 1 and Fig. 2*A*). The equilibrium binding constant is consistent with our previous ITC assays for a preQ_1_-I_III_ riboswitch from *E. cloacae*, which had a *K*_D_ of 72 nM (27).

**Table 1:** Isothermal calorimetry measurement of preQ_1_ affinity for the WT *Eco* riboswitch and linker variants

*Eco* *Sequence*	*K*_*D*_*(nM)*	*N (no. of sites)*	*ΔH (kcal mol*^*–1*^*)*	*–TΔS (kcal mol*^*–1*^*)*	*ΔG (kcal mol*^*–1*^*)*	*K*_*rel*_^a^
*WT 36-mer*	57.9 ± 1.5	0.9	−23.1 ± 0.3	13.2 ± 0.3	−9.9 ± 0.0	-
*35-mer*	33.4 ± 1.9	0.8	−32.9 ± 1.6	22.7 ± 1.6	−10.2 ± 0.0	1.7
*34-mer*	14.0 ± 1.6	1.0	−26.0 ± 0.1	15.3 ± 0.2	−10.7 ± 0.1	4.2
*33-mer*	30.2 ± 1.3	0.9	−28.8 ± 0.5	18.5 ± 0.5	−10.3 ± 0.0	1.9
*30-mer*	51.9 ± 1.8	0.9	−25.7 ± 0.1	15.6 ± 0.1	−9.9 ± 0.0	1.1

a Calculated as *K*_D, mutant_/*K*_D, WT_.

Despite this strong ligand affinity, cocrystals of the WT *Eco* riboswitch and other type III riboswitch sequences were refractory to crystallization (data not shown). The covariation model suggests that three positions of the SDS, 5′-GGA-3′, form the pseudoknot helix P2 (9). Directly preceding the P2 pseudoknot are 4 to 5 strongly conserved adenine nucleotides (*i.e.*, the polyA region) connected to the base of the P1 helix *via* a linker having variable length and sequence (Fig. 1*B*). Based on available structures for class I type I and II riboswitches from *Can* (26), *Tte* (13, 14, 21, 22), and *Bsu* (15, 33), this conserved polyA region was hypothesized to mediate a series of A-amino kissing interactions that form a crossover element in the P1 minor groove that stabilizes the fold and forms the floor of the binding pocket. We hypothesized that we could promote crystal formation by shortening the poorly conserved linker region to remove flexible nucleotides without weakening preQ_1_ affinity.

To test this hypothesis, we generated three mutants with successively longer nucleotide deletions in the linker and tested the preQ_1_ binding of each. The WT *Eco* riboswitch has a linker sequence of 5′-GGUUAAUC-3′ (Fig. 2*A*). A single G deletion (*i.e.*, *Eco* 35-mer with a linker sequence of 5′-GUUAAUC-3′) enhanced the *K*_D_ to 33.4 ± 1.9 nM (Table 1 and Fig. 2*B*). A ΔGG double deletion mutant (*i.e.*, *Eco* 34-mer with a linker sequence of 5′-UUAAUC-3′) had a markedly improved affinity with an average *K*_D_ of 14.0 ± 1.6 nM (Table 1 and Fig. 2*C*). Finally, the triple deletion mutant ΔGGU (*i.e.*, *Eco* 33-mer with a linker sequence of 5′-UAAUC-3′) had higher affinity relative to WT with an average *K*_D_ of 30.2 ± 1.3 nM, which was comparable to the 35-mer ΔG single mutant (Table 1 and Fig. 2*D*). Thus, shortening the poorly conserved linker indeed improved the affinity of preQ_1_-I_III_ riboswitches for preQ_1_.

### The *Eco* 30-mer crystallization construct tightly binds preQ_1_

Of the three linker deletion mutants having improved affinity for preQ_1_, only the 34-mer double mutant readily crystallized. However, crystals were sensitive to cryoprotection even after dehydration (34) and showed no appreciable diffraction (data not shown). As such, we returned to the strategy of altering the nonconserved L3 linker sequence that joins P1 and P2.

We further shortened the linker preceding the polyA sequence to four nucleotides and mutated the sequence to match previous preQ_1_-I cocrystal structures at the P1-L3 turn, which involves a sharp bend in the backbone (13, 14, 15, 21, 22, 33). We changed the first two nucleotides in the linker region to uridine and cytidine that are similar to the WT sequence of the type II *Tte* riboswitch (13, 14, 21, 22). We next added an adenine, which we hypothesized would interact with the minor groove of P1, followed by uracil to avoid an unnatural extension of the conserved L3 polyA sequence (Fig. 1*B*). Placement of adenines in the proper register is known to be important for minor-groove stabilization at P1. Moreover, the binding-pocket floor of type I and II riboswitches adopts a quintuple-base transition motif (35) that engages minor-groove contacts from two conserved adenines in the L3 loop (13, 14, 15, 21, 22, 26, 33). Thus, we hypothesized that the type III pocket floor contains a comparable base-transition module. Finally, we removed one base pair from the P1 stem to promote crystal packing typical of other preQ_1_-I subtypes (13, 14, 15, 21, 26).

Importantly, our 30-mer *Eco* construct retained the highly conserved, pyrimidine-rich stem loop region predicted to compose the aptamer domain and part of the gene-regulatory P2 helix. The crystal-promoting mutations were confined to the nonconserved region of the consensus model and away from the binding pocket and SDS expression platform, providing confidence that the construct would provide atomic-level details of preQ_1_ recognition and gene regulation (Fig. 1*B*).

Consistent with this possibility, the 30-mer *Eco* riboswitch sequence showed marginally better preQ_1_ binding compared to WT with an average *K*_D_ of 51.9 ± 1.8 nM (Table 1 and Fig. 2*E*). This binding constant suggests that the length and sequence of the linker region allows metabolite binding similar to WT but is not as ideal as the double-deletion 34-mer (Table 1 and Fig. 2, *A* and *C*), which does not appear to occur naturally because binding could be too tight to allow effective gene regulation. Nonetheless, under low-salt conditions, the *Eco* 30-mer produced well-diffracting crystals.

### Mn^2+^ promotes phasing, quality of the refined model, and the pseudoknot fold

Despite the prediction that the *Eco* riboswitch would adopt the same H-type pseudoknot fold as other class I preQ_1_ riboswitches (9), molecular replacement was unsuccessful for phasing due to dissimilarities in the unusually small type III pyrimidine-rich aptamer compared to those of known type I and II preQ_1_ riboswitches (Fig. 1*B*). To circumvent this issue, we crystallized the 30-mer *Eco* crystal with MnCl_2_ for SAD phasing. We previously demonstrated for the *Tte* riboswitch that Mn^2+^ ions can act as a proxy for Mg^2+^ through coordination at magnesium-binding sites (21). Indeed, Mn^2+^ and Mg^2+^ bind with nearly identical octahedral coordination geometry (36), although Mn^2+^ prefers inner sphere coordination at purine nitrogen atoms compared to Mg^2+^ (37). In the 30-mer *Eco* riboswitch, the ion substructure comprises four site-bound Mn^2+^ ions per asymmetric unit (described below). To our knowledge, this is this first report of an RNA structure solved using Mn^2+^ SAD phasing.

The structure of the 30-mer preQ_1_-I_III_ *Eco* riboswitch was refined to 2.30 Å resolution. The *R*_work_/*R*_free_ values were 20.5%/25.2% with bond and angle RMS deviations from ideality of 0.003 Å and 0.524˚ (Table 2). Two RNA chains were modeled in the asymmetric unit, which varied in completeness. Chain A was well defined by electron density, whereas chain B had a break in the linker connecting P1 and L3 (Fig. S1*A*); chain B also lacked density for the C12 base (Fig. S1*B*). In the P1 helix, position U1 does not form the expected canonical base pair with A19. Instead, U1 is disordered, causing A19 to engage in a crystal contact with the Hoogsteen edge of A19 from a symmetry-related molecule (Fig. S1*C*).

**Table 2:** X-ray data reduction & refinement statistics

Riboswitch sample	*Eco* 30-mer
Data collection	
Space group	*P* 2
Cell dimensions	
*a*, *b*, *c* (Å)	46.7, 32.3, 51.2
α = γ, β (°)	90.0, 96.9
Wavelength (Å)	1.85
*R*_*p.i.m.*_*(%)*^a^	2.4 (27.0)
*I*/σ(*I*)	16.7 (2.8)
Completeness (%)	98.1 (97.9)
Redundancy	22.0 (16.3)
CC1/2	0.99 (0.92)
FOM (FOM)^b^	0.30 (0.62)
Refinement	
Resolution (Å)	36.52–2.30 (2.38–2.30)
No. of reflections	12,803
*R*_work_/*R*_free_ (%)	20.5/25.2
No. of atoms	
RNA	1696
Ligand/Ion	48
Water	13
*B*-factors (Å^2^)	
RNA	63.7
Ligand/Ion	52.2
Water	58.5
r.m.s. deviations	
Bond lengths (Å)	0.003
Bond angles (°)	0.524

a *R*_precision-__indicating merging__R-value_ = $\frac{\sum_{\text{hkl}}\sqrt{\frac{1}{N-1}}\sum_{i=1}^{N}|I\left(\text{hkl}\right)-<I\left(\text{hkl}\right)>|}{\sum_{\text{hkl}}\sum_{i=1}^{N}I\left(\text{hkl}\right)}$, where N is the redundancy of the data and ⟨I(hkl)⟩ is the average intensity.

b Figure of merit from SAD phasing (after density modification).

**Figure 3:**
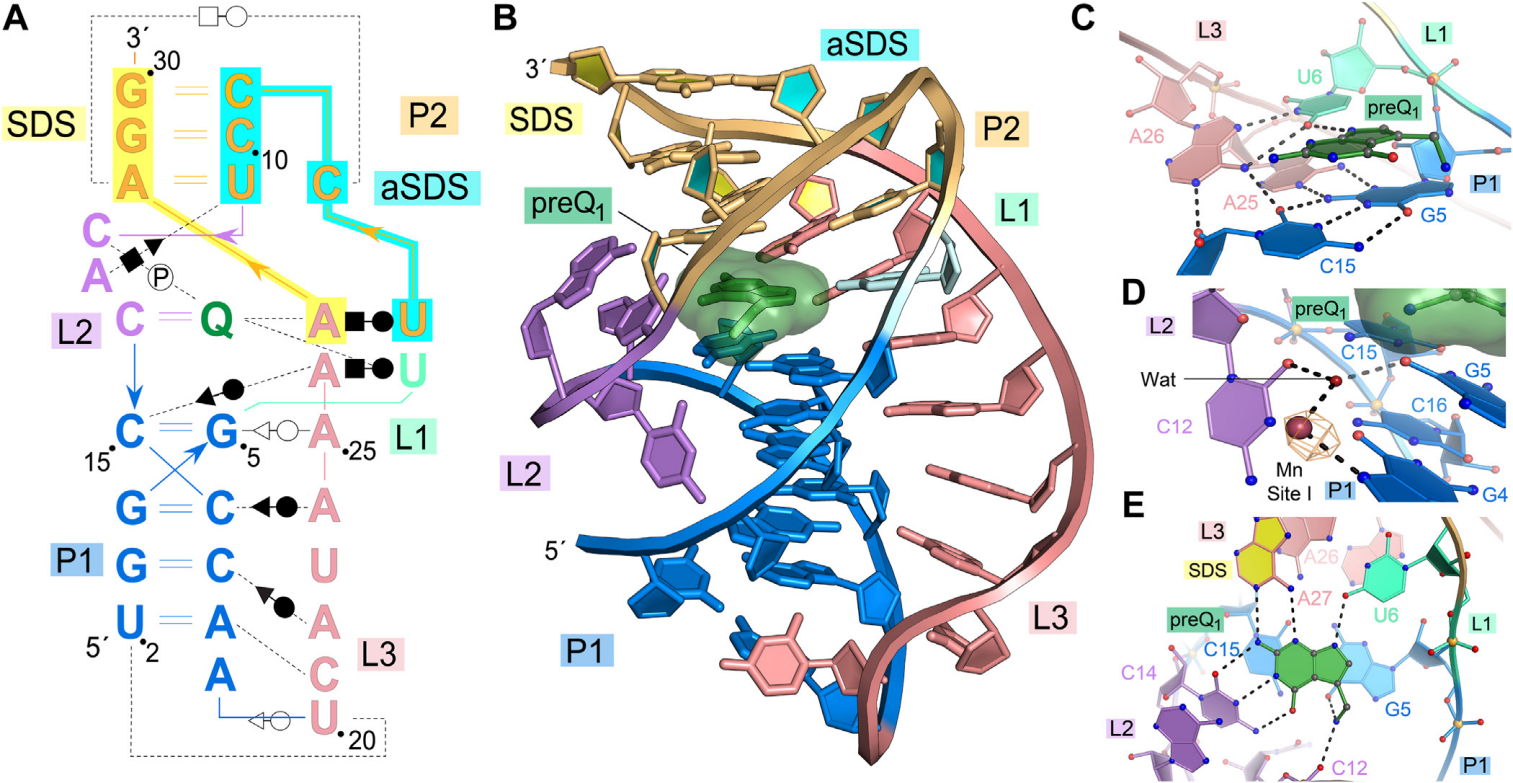
**Schematic view, ribbon diagram, and close-up views of the class I type III preQ**_**1**_**(preQ**_**1**_**-I**_**III**_**) riboswitch from *E. coli*.***A*, secondary structure of the *Eco* crystallization construct. Positions are colored according to pseudoknot pairing and loop regions observed in the co-crystal structure; preQ_1_ is shown as Q (*green*). Interactions between specific nucleotides based on the crystal structure are annotated with Leontis-Westhof symbols (66). *B*, ribbon diagram of the *Eco* riboswitch based on the co-crystal structure. *C*, close-up view of the preQ_1_-binding pocket floor formed by a quintuple base transition motif comprising two base triples: a planar triple at G5-C15•A25 and a transition triple at C15•A26•U6. *D*, site I Mn^2+^ ion in stem P1 shown inside anomalous difference Fourier electron density contoured at 6.5σ. PreQ_1_ (surface model) binds atop the nearby G5-C15 base pair of stem P1. *E*, overview of the preQ_1_-binding pocket showing preQ_1_ interacting with A27 of the SDS (emphasized by *yellow*-filled nucleotide rings).

The *Eco* preQ_1_-I_III_ riboswitch folds as an extraordinarily compact H-type pseudoknot characterized by numerous noncanonical base interactions (Fig. 3, *A* and *B*). The fold involves a short, conserved P1 helix that transitions through a single-nucleotide L1 loop at U6 into the P2 (pseudoknot) helix, which comprises canonical and noncanonical base pairs. At only three nucleotides, the L2 loop is especially short and the nucleobase of each loop points toward the bound preQ_1_ metabolite. Following the 3′-end of helix P1, the backbone makes a sharp bend into loop L3, which exhibits a series of contacts including A-amino kissing interactions (Fig. S2, *A* and *B*) that are functionally equivalent to those seen in type I and type II preQ_1_-I riboswitches (21, 26). Position A25 and A26 of the A-rich loop complete a quintuple-base transition motif (35) that links the metabolite-binding pocket to the pocket floor, consistent with our hypothesis (above). The remarkable compactness of the fold is underscored by the presence of nine base triples, which exceeds the seven canonical (Watson-Crick) base pairs (Fig. 3*A*).

### Mn^2+^ coordinates the major-groove edge of a conserved guanine in helix P1

Each of the two *Eco* riboswitch molecules in the asymmetric unit contained four site-bound Mn^2+^ ions at different locations (Fig. S3*A*). In anomalous difference Fourier maps, all Mn^2+^ ions exhibited strong anomalous scattering with peak heights of 6.5σ (site I), 8.5σ (site II), 8.5σ (site III), and 6.0σ (site IV). At site I, the ion coordinates N7 of G4 in helix P1 (Fig. 3*D*), consistent with findings that Mn^2+^ strongly coordinates N7 of guanine nucleotides in the major groove (21, 37). The consensus model for preQ_1_-I_III_ riboswitches indicates that three G-C base pairs are highly conserved in the P1 stem that contains G5, which resides in the floor of the binding pocket (Figs. 1*B* and 3*C*). These observations suggest that Mg^2+^ binds at this position and appears to stabilize the top of the stem to facilitate interaction between the preQ_1_ exocyclic amine and the G5 O6 keto group (Fig. 3, *C*–*E*).

In contrast to site I, Mn^2+^ ions at sites II, III, and IV do not appear to contribute to the stabilization of the overall fold. Instead, these ions engage in crystal contacts. Mn^2+^ at sites II and III coordinate N7 of G30 in chain A and a nonbridging oxygen of the phosphate backbone at C9, which is donated by the crystallographically related chain B (Fig. S3*B*). Intermolecular Mn^2+^ coordination connects the flush-end stack between the C9-G30 Watson-Crick base pairs that cap the P2 stems of chains A and B. The Mn^2+^ ion at site IV also mediates a crystal contact between nonbridging oxygens of A18 and A19 in chains A and chain B, which form inner-sphere ion contacts (Fig. S3*C*). Taken together, these results explain the requirement of Mg^2+^ or Mn^2+^ ions for crystallization of the *Eco* preQ_1_-I_III_ riboswitch.

### Conservation of class I preQ_1_ riboswitch folds and metabolite-binding pockets

**Figure 4:**
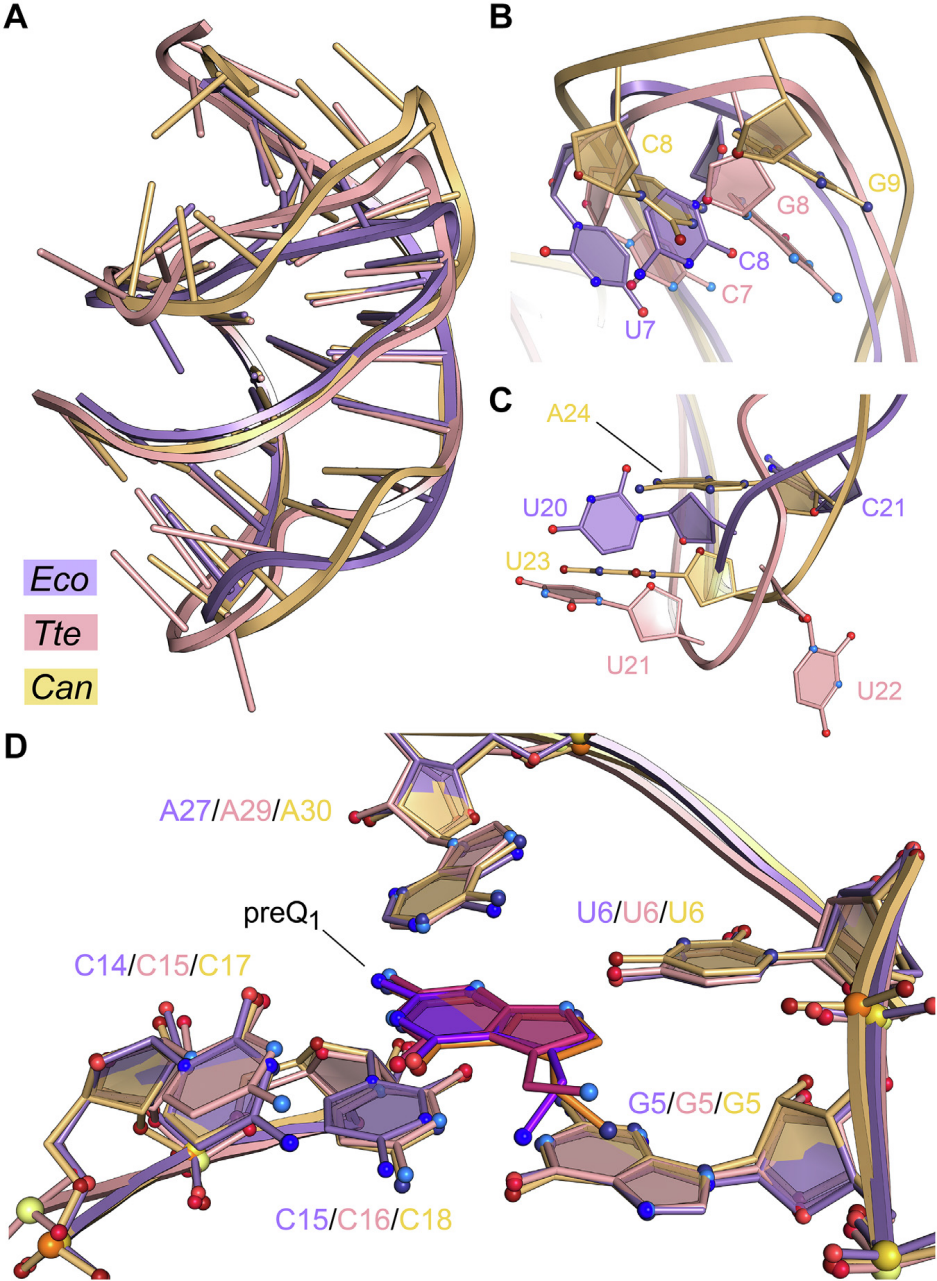
**Superposition of the *Eco* type III riboswitch with the type I *Can* riboswitch and type II *Tte* riboswitch.***A*, ribbon diagram of chain A superimposed on all paired atoms of the *Eco* (*purple*), *Tte* (13) (*salmon*), and *Can* riboswitches (*gold*) (26). Close-up views of (*B*) *Eco* riboswitch U7 and C8, which produce a tight bend facilitated by the pyrimidine-rich sequence in this region. *C*, P1-L3 transition showing closest agreement between the *Eco* riboswitch and the *Can* riboswitch coordinates. *D*, α-site preQ_1_ binding pocket in *Eco*, *Tte*, and *Can* riboswitches.

The *Eco* riboswitch superimposes with representative chains from the *Can* preQ_1_-I_I_ and *Tte* preQ_1_-I_II_ riboswitch cocrystal structures with average rmsd values of 1.51 Å and 1.69 Å for all paired atoms (Fig. 4*A*). The largest conformational differences localize to helix P2 where the type I *Can* riboswitch has three additional nucleotides at positions 9 to 11 that expand the backbone of the pseudoknot loop (Fig. 4*B*, *yellow*) to accommodate a second preQ_1_ metabolite (*i.e.*, the β site). By contrast, the type III *Eco* riboswitch adopts a very tight pseudoknot loop that allows binding of only a single metabolite (Fig. 4*B*, *purple*). The type II *Tte* riboswitch also binds one metabolite but has a loop of intermediate size relative to *Eco* and *Can* (Fig. 4*B*, *pink*).

The P1-to-L3 turn also exhibits different conformations among the riboswitches (Fig. 4*C*). Here, the *Eco* riboswitch backbone conformation most closely resembles that of the *Can* riboswitch, which shows a one-to-one correspondence for each nucleotide (*i.e.*, no insertions or deletions) although the sequences are different (*i.e.*, 5′-UCAUAA-3′ for the *Eco* riboswitch and 5′-UAAAAA-3′ for the *Can* riboswitch). In general, the *Eco* riboswitch makes tighter turns with fewer nucleotides than its type I and type II subclass counterparts.

Comparing the *Eco*-binding pocket to other class I riboswitches reveals a striking similarity with the α-site–binding pocket of the type I *Can* and type II *Tte* riboswitches (21, 26) (Fig. 4*D*). Each riboswitch has a highly conserved cytidine in L2 acting as a specificity base that reads out the Watson-Crick-face of preQ_1_ through canonical hydrogen bonding. Meanwhile, highly conserved adenines from L3 and uridines from L1 recognize the metabolite *via* its minor-groove edge equivalent (Fig. 4*D*). The only difference among the three binding sites is the orientation of the flexible methylamine group (Fig. 4*D*), which is likely caused by differences in the local charge environment. In particular, the *Eco* riboswitch forms salt-bridge interactions between the charged methylamine and the phosphate backbone at position 12 (Fig. 3*E*), and these interactions are absent in the *Can* and *Tte* riboswitch structures (21, 26).

Another distinct trait of the *Eco* type III riboswitch is a direct interaction between the first position of the SDS, A27, and preQ_1_ (Fig. 3*E*). In *E. coli* bacteria, the SDS consensus is 5'-(A/C)(A/U)GGA(A/G)AA (38), which closely matches both the consensus of type III riboswitch 3′-tails (5′-AAGG, Fig. 1*B*) and the *Eco* riboswitch here. This observation provides a direct link between effector binding and gene regulation that is not a feature of the other preQ_1_-I subtypes (21, 26). Similarly, the SDS is not involved in metabolite recognition by either class II or class III preQ_1_ riboswitches (30, 31), although for the preQ_1_-II riboswitch, the first base of the SDS lies in the pocket floor directly beneath the metabolite (31, 39).

### Type III riboswitches sequester more of the SDS in the P2 helix pseudoknot

**Figure 5:**
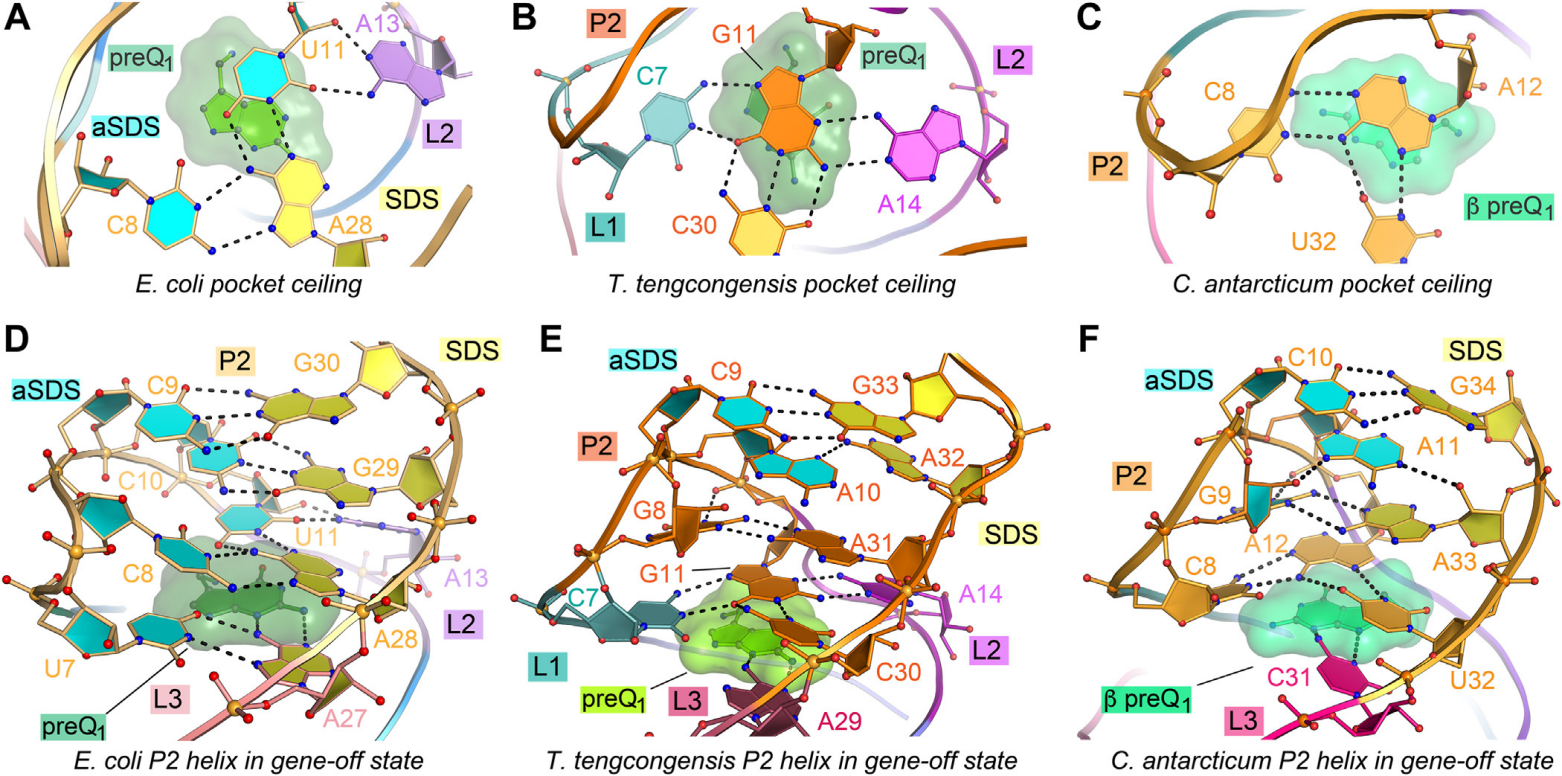
**Comparison of representative class I riboswitch types at binding pocket ceilings and modes of Shine-Dalgarno sequence sequestration in helix P2.***A*, close-up top view of *Eco* (type III) binding pocket ceiling. SDS nucleotide A28 and anti-SDS nucleotides C8 and U11 are highlighted as *yellow* and *cyan* base rings; preQ_1_ is in *green* with a semitransparent surface. *B*, *Tte* (type II)-binding pocket ceiling (PDB 6vui) (21). *C*, *Can* (type I) binding pocket ceiling (PDB 8fb3) (47). *D*, overview of the *Eco* riboswitch expression platform. The SDS is sequestered by binding pocket interactions, the pocket ceiling, and helix P2, which engage in canonical and noncanonical interactions with the anti-SDS. The P2 helix of (*E*) *Tte* and (*F*) *Can* riboswitch with the first two positions of the SDS sequestered by interactions with the anti-SDS.

In the type III *Eco* riboswitch, the SDS extends into the pocket ceiling, such that the second position of the SDS, A28, is sequestered in a base quartet that stacks atop preQ_1_ (Fig. 5*A*). This feature is reminiscent of the *Tte* riboswitch (Fig. 5*B*) but differs from the base-triple ceiling of the *Can* riboswitch, which sits above the β-site preQ_1_ (Fig. 5*C*). The core of the *Eco* riboswitch ceiling is a base triple that involves C8 and U11 from the anti-SDS (*i.e.*, the strand complementary to the SDS) and A28 of the SDS (Fig. 5*A*). Additionally, the Watson-Crick face of A13 from L2 contacts the minor groove of U11 (Fig. 5*A*). In the *Tte* riboswitch, the core of the ceiling includes a G11-C30 canonical pair that forms a base quadruple in which C7(+) pairs with the Hoogsteen edge of G11, and the WC face of A14 of loop L2 interacts with the minor-groove edge of G11 (Fig. 5*B*). The latter interaction resembles U11 and A13 of the *Eco* riboswitch (Fig. 5*A*). Notably, the *Tte* riboswitch structure determined from crystals grown in the absence of preQ_1_ (13, 21) shows A14 occupying the binding pocket where it functions as a gatekeeper residue (21). *Tte* A14 appears to be analogous to A13 in *Eco*, suggesting that *Eco* A13 may play a similar gatekeeping role.

The *Eco* riboswitch SDS extends further into the end of P2 relative to its type I and II counterparts (Fig. 5, *D*–*F*). The first two positions of the SDS, A27 and A28, engage in base triple interactions, while the final two guanines, G29 and G30, engage in *cis* Watson Crick interactions with C10 and C9 (Fig. 5*D*). Watson Crick pairing between U11-A28, C10-G29, and C9-G30 was inferred from the covariation model (Fig. 1*B*) built during the initial classification of this subtype (9). However, the base triple interactions involving U7•A27•preQ_1_ and C8•A28-U11 were only apparent in the structure shown here (Fig. 5*D*). Collectively, these and other similar interactions explain the unusual pyrimidine-rich composition of the aptamer loop that extends from nucleotides 6 through 12 of the *Eco* riboswitch (Fig. 3*A*).

The SDS sequestration observed in the *Eco* riboswitch is quite strong compared to other preQ_1_-I riboswitch subtypes, in which only the first two SDS nucleotides are integrated into pseudoknot folds (Fig. 5, *E* and *F*). Indeed, the *Tte* preQ_1_-I_II_ riboswitch strongly sequesters G33 *via* a Watson-Crick interaction with C9, whereas the first position, A32, has only a single hydrogen bond to A10 of the anti-SDS (Fig. 5*E*). Likewise, the *Can* preQ_1_-I_I_ riboswitch firmly sequesters the second SDS position, G34, *via* a strong Watson-Crick pair with C10. However, A33 in the first position of the SDS engages its Watson-Crick face with the sugar edge of G9 and the Watson-Crick face of A11 through three hydrogen bonds (Fig. 5*F*) (26).

In addition to differences in SDS sequestration, the overall architecture of the P2 helix in the *Eco* riboswitch differs from the type II *Tte* and type I *Can* riboswitches. Relative to the *Eco* riboswitch, both *Tte* and *Can* riboswitches have a less severe bend of the P2-helix hairpin due to the presence of a guanine (G8 in *Tte* and G9 in *Can*) that lies directly above the pocket ceiling where the N1 imino group can interact with a nonbridging phosphate oxygen of the backbone in P2 (Figs. 4*B* and 5, *D*–*F*). The minor-groove of this guanine also interacts with the Watson-Crick face of a cross-strand adenine (A31 in *Tte* and A33 in *Can*) (Fig. 5, *E* and *F*). Another adenine (A10 in *Tte* and A11 in *Can*) stacks between this noncanonical G•A pair and the terminal G-C pair and donates a single hydrogen bond to the first position of the SDS (A32 in *Tte* and A33 in *Can*) (Fig. 5, *E* and *F*). By contrast, the *Eco* riboswitch, like all preQ_1_-I_III_ sequences (9), has a pyrimidine-rich loop (Fig. 1*B*) that cannot form these purine-mediated contacts (Fig. 5*D*) and thus has a more compact pseudoknot with more canonical pairing that together are predicted to render the *Eco* riboswitch more resistant to ribosome helicase activity.

### PreQ_1_-I_III_ riboswitches selectively sense preQ_1_ over preQ_0_

**Figure 6:**
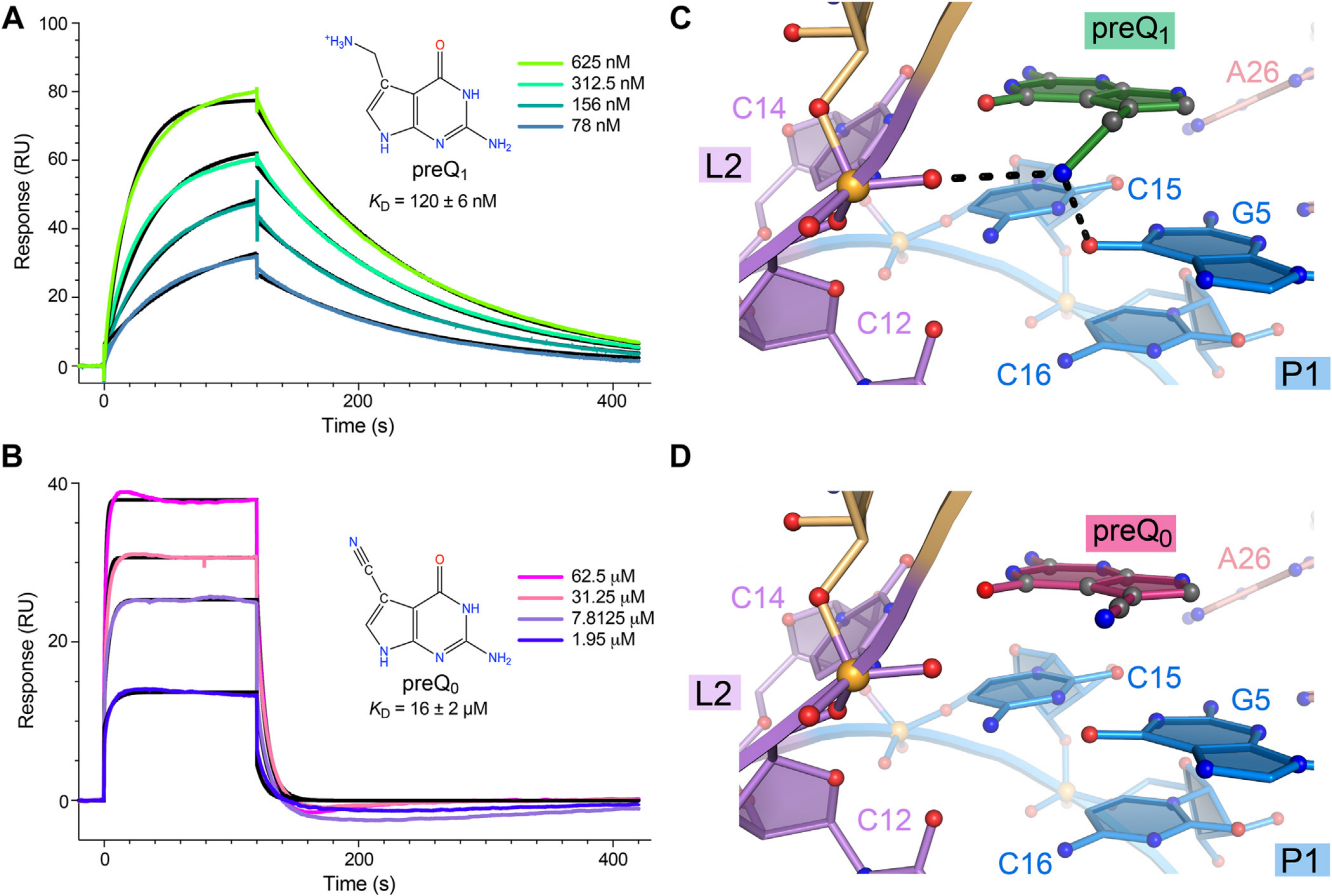
**SPR kinetic analysis of preQ**_**1**_**and preQ**_**0**_**metabolite binding to the WT *Eco* riboswitch.***A*, representative SPR sensorgrams showing preQ_1_ association and disassociation with the WT *Eco* riboswitch. *B*, representative SPR sensorgrams showing preQ_0_ association and dissociation with the WT *Eco* riboswitch. *C*, close-up view of the preQ_1_ 7-aminomethyl moiety that donates hydrogen bonds to the nonbridging phosphate oxygen of C12 and O6 of G5. *D*, hypothetical model of preQ_0_ binding to the *Eco* riboswitch based on superposition of the precursor metabolite on the pyrrolopyrimidine moiety of the experimentally derived preQ_1_ model. Kinetic constants are provided in Table 3.

Based on the cocrystal structure, we hypothesized that the affinity of preQ_1_-I_III_ riboswitches for preQ_0_—the metabolic precursor of preQ_1_ (Fig. 1*A*)—would be lower than that for preQ_1_ due to geometric and electronic differences between the nitrile and the methylamine groups at the C7 position of the pyrrole ring (Fig. 6, *A* and *B*). This possibility is intriguing since proteobacteria like *E. coli* lack *queT* transporter genes and instead utilize *yhhQ* genes to salvage preQ_1_-related molecules from the environment. Notably, a recent study demonstrated that *yhhQ* genes encode a protein that imports preQ_0_ with preference over preQ_1_ (28). Moreover, the *Tte* preQ_1_-I_II_ riboswitch, which has a binding pocket that is highly homologous to the *Eco* riboswitch (Fig. 4*D*), was shown to have relatively high affinity for preQ_0_ (*K*_D_ of 35 nM; 17-fold lower than that for preQ_1_) (13). Considering these factors, we investigated preQ_0_ as a natural metabolite for class I type III preQ_1_ riboswitches, such as the *Eco* riboswitch studied here.

We first used SPR to measure binding kinetics of the WT *Eco* sequence for preQ_1_. The results revealed that the WT *Eco* riboswitch binds preQ_1_ with an apparent *K*_D_ of 120 ± 6 nM, which is consistent with ITC measurements (Tables 1 and 3 and Figs. 6*A* and 2*A*). The WT *Eco* riboswitch *k*_on_ and *k*_off_ for preQ_1_ is 66.1 × 10^3^ M^–1^ s^–1^ and 7.9 × 10^-3^ s^–1^, corresponding to *t*_1/2_ of 88.4 s. The *Eco* riboswitch *k*_on_ is similar to that for the *Tte* riboswitch (*k*_on_ 77.7 × 10^3^ M^–1^ s^–1^) (13), but its *t*_1/2_ is shorter due to its 50-fold difference in *k*_off_. This difference in dissociation rate is likely associated with the more stable fold of the thermophilic *Tte* riboswitch than that of *Eco*.

**Table 3:** SPR measurements of preQ_1_ and preQ_0_ binding kinetics for WT *Eco* and mutants

*Eco* *Sequence*	*Ligand*	*k*_*on*_*x 10*^*3*^*(M*^*−1*^ s^*−1*^*)*	*k*_*off*_*x 10*^*–3*^*(s*^*–1*^*)*	*K*_*D*_*(nM)*	*t*_*1/2*_*(s)*	*K*_*rel*_^a^
*WT 36-mer*	preQ_1_	66.1 ± 2.2	7.93 ± 0.1	120 ± 6	88.4	-
*WT 36-mer*	preQ_0_	9.8 ± 0.2	155.3 ± 1.9	15,970 ± 2260	4.5	133
*G6DAP/C16U*	preQ_1_	–	–	77,390 ± 5880	–	645
*U7C*	preQ_1_	–	–	4,220,000 ± 33,000	–	35,167
*A33Purine*	preQ_1_	–	–	4,706,000 ± 276,000	–	39,217
*C15U*	preQ_1_	–	–	7,682,000 ± 206,000	–	64,017

a Calculated as *K*_D, mutant_/*K*_D, WT_.

The binding kinetics for preQ_0_ binding to the *Eco* riboswitch showed lower affinity for preQ_0_ than preQ_1_ as demonstrated by a slower *k*_on_ (9.8 × 10^3^ M^–1^ s^–1^) and faster *k*_off_ (155 × 10^–3^ s^–1^) for preQ_0_ that corresponds to a *t*_1/2_ of 4.5 s (Fig. 6*B*). Accordingly, the apparent *K*_D_ of the *Eco* riboswitch for preQ_0_ was 15.9 ± 2.3 μM, which represents nearly a 133-fold loss in affinity compared to preQ_1_ (Table 3). Although the *Tte* riboswitch had a comparable *k*_on_ for preQ_0_ (9.8 × 10^3^ M^–1^ s^–1^ *versus* 6.5 10^3^ M^–1^ s^–1^), the *Eco k*_off_ value was 44-fold slower (2.22 × 10^–4^ s^–1^), resulting in a t_1/2_ of 54 min. These results demonstrate that the type III *Eco* riboswitch strongly discriminates between preQ_0_ and preQ_1_ largely through the relatively fast dissociation of the preQ_0_ metabolite (Fig. 6*B*).

To provide insight into the mode of preQ_0_ discrimination, we next considered how the preQ_0_ metabolite would fit into the binding pocket in the *Eco* cocrystal structure. The flexible 7-aminomethyl group of preQ_1_ lies in a cleft between L2 and P1 where it acts as a hydrogen bond donor to O6 of G5 in the binding pocket floor and a nonbridging phosphate group of C12 in loop L2 (Fig. 6*C*). Although our experiments suggest that preQ_0_ can be accommodated in the binding pocket, the nitrile group at the 7-position of preQ_0_ is unprotonated (Fig. 6*B*), which precludes the hydrogen bond formation required for favorable binding to and specificity for preQ_1_ (Fig. 6*C*). The linear *sp* geometry of the preQ_0_ nitrile moiety likely causes a steric clash with the backbone based on modeling (Fig. 6*D*). These factors plausibly contribute to the 6.7-fold slower binding and faster off-rate for preQ_0_ than to preQ_1_ (Table 3).

### Mutation of key positions validates the observed mode of preQ_1_ binding

Based on the structural similarity between the binding pockets of the *Eco* and *Tte* riboswitches (13), which both bind a single metabolite (Fig. 4*D*), we generated a series of homologous mutations that were probed by SPR to examine how conserved metabolite-binding nucleotides in the *Eco* riboswitch affect preQ_1_ recognition.

**Figure 7:**
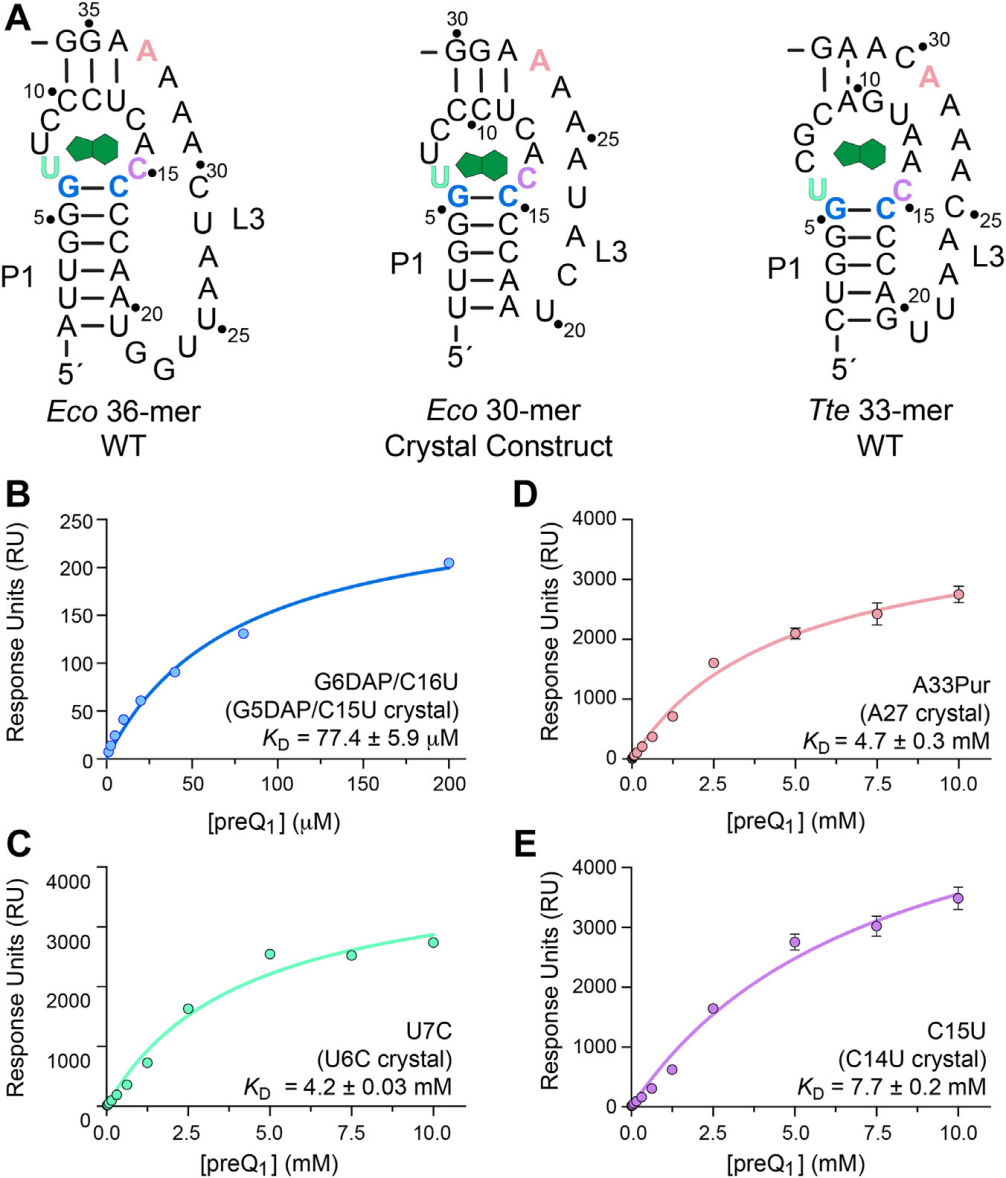
**SPR equilibrium binding analysis of *Eco* preQ**_**1**_**-I**_**III**_**riboswitch mutants.***A*, schematic diagrams of the WT *Eco* 36-mer (*left*), *Eco* crystal construct 30-mer (*center*), and WT *Tte* 33-mer; preQ_1_ is shown in *green*. Positions colored *blue*, *green*, *pink*, and *purple* correspond to mutations made for this study that are homologous to those previously made in the *Tte* riboswitch (13). Positions are colored based on regions of the H-type pseudoknot defined in Figure 3*B*. Mutants are numbered according to the WT sequence with positions in the crystallization construct shown in parentheses. *B*, *Eco* G6DAP/C16U double mutant binding response to preQ_1_. *C*, U7C mutant. *D*, A33Purine mutant. *E*, C15U mutant. Binding constants and standard errors are provided in Table 3. All measurements were made in triplicate.

We first analyzed the importance of the G6-C16 base pair in the WT *Eco* sequence (numbered G5-C15 in the cocrystal structure; Fig. 7*A*). This canonical pair contributes to the binding pocket floor (Figs. 3*E* and 4*D*) and is invariant across all preQ_1_-I subtypes (Fig. 1*B*) (9). This conservation is likely associated with the interaction of the preQ_1_ methylamine group with O6 of G5, as observed in our structure (Fig. 3*E*), or with N7 of G5 as observed in previous preQ_1_-I riboswitch structures (13, 15, 21, 33). To change the O6 keto oxygen to an exocyclic amine while maintaining Watson-Crick–like base pairing, we generated a double mutant with G6 mutated to 2,6-diaminopurine (DAP) and C15 mutated to uracil. An analogous G5DAP/C16U mutant was made previously in the *Tte* riboswitch, which has a structurally homologous base pair (Fig. 4*D*) (13). As predicted, replacing the O6 keto group with an exocyclic amine reduced preQ_1_ affinity by around 645-fold compared to WT (*K*_D_ of 77.4 ± 5.9 μM *versus* 0.12 μM for WT) (Fig. 7*B* and Table 3). Notably, this loss in affinity was similar to the 981-fold reduction observed for the G5DAP/C16U mutant in the *Tte* riboswitch (13). The *Eco* G6DAP/C16U mutation maintains the minor-groove edge features of the WT riboswitch that participate in the A-amino kissing interaction with A25 and A26 (Fig. 3*C*), but this substantially reduced affinity highlights the role of the O6 keto group of G5 as an important determinant of preQ_1_ specificity and affinity that likely discriminates against other purine-like metabolites.

We next mutated U7 to cytosine (U6C in the cocrystal structure, Fig. 7*A*), which would disrupt the single hydrogen bond with the minor-groove edge equivalent of preQ_1_ (Fig. 3*E*). The U7C mutant indeed reduced affinity for preQ_1_, with a 35,000-fold change compared to WT (*K*_D_ of 4.2 ± 0.3 mM; Figure 7*C* and Table 3). The U6C mutation was predicted to disrupt only a single hydrogen bond to preQ_1_, but closer inspection of the U6 Watson-Crick face in the crystal structure reveals an interaction with the Hoogsteen edge of A26, which forms part of the quintuple-base transition motif that is part of the binding pocket floor (Fig. 3*C*). The analogous mutation in the *Tte* riboswitch also showed poor affinity with an estimated *K*_D_ >274 μM, representing an approximate 134,000-fold decrease in binding (13). The base equivalent of the *Tte* riboswitch, U6, also makes a Hoogsteen-edge interaction with an adenine at position 28 (not shown), which would be disrupted by a U-to-C mutation. Together, these observations suggest that the quintuple-base transition motif involves U6 to form a stable floor of the binding pocket and plays a key role in connecting the aptamer to the P1 helix, which is likely essential to stabilize the pseudoknot fold.

Additional recognition of the preQ_1_ minor-groove-edge equivalent is achieved by A33 (Fig. 7*A*), which is numbered A27 in the cocrystal structure (used here for convenience). Specifically, N1 and N6 groups of A27 hydrogen bond to N2 and N3 of preQ_1_ (Fig. 3*E*). The A27 Hoogsteen edge also interacts with the Watson-Crick face of U7 (Fig. 5*D*), similarly to the U6•A26 interaction directly below it (described above and Fig. 3*C*). To ameliorate the loss of affinity caused by disruption of Hoogsteen edge interactions in the U6C mutant, we generated an A27Pur (purine) mutant to partially maintain the U7•A27 interaction while weakening preQ_1_ recognition by removing only the A27 exocyclic amine. The A27Pur mutant had a 39,000-fold reduction in affinity relative to WT (*K*_D_ of 4.7 ± 0.3 mM; Fig. 7*D* and Table 3) that is far larger than the ∼9000-fold increase observed for the equivalent A29Pur mutation in the *Tte* riboswitch (13). This unexpectedly large decrease in preQ_1_ binding for A27Pur suggests that for the *Eco* riboswitch, the Hoogsteen interaction of A27 with U7 is critical to lock this base into the correct geometry needed to recognize the minor groove of preQ_1_ in preQ_1_-I_III_ riboswitches.

Last, we generated the C15U mutant (C14U in the cocrystal structure, Fig. 7*A*) that represents the specificity base for preQ_1_ (Fig. 3 *E*). The consensus model for type III riboswitches indicates 97% conservation at C15, which is hypothesized to recognize preQ_1_ through Watson-Crick readout of the ligand’s guanine-like face, akin to the *Tte* and *Bsu* type II riboswitches (9, 15, 33) and the *Can* type I riboswitch α site (26). Mutation of the analogous position in the *Bsu* preQ_1_-I_II_ riboswitch from *B. subtilis* essentially abolished binding (8). Similarly, the *Eco* C15U mutant had the weakest preQ_1_ recognition of the three mutants (*K*_D_ of 7.7 ± 0.2 mM; 64,000-fold reduction relative to WT) (Fig. 7*E* and Table 3). Together, these results indicate that the determinants of preQ_1_ recognition by the *Eco* riboswitch resemble that of the *Tte* preQ_1_-I_II_ riboswitch, but the *Eco* riboswitch has a lower tolerance for mutations in the binding pocket.

### *Eco* riboswitch control of gene regulation is sensitive to mutations

**Figure 8:**
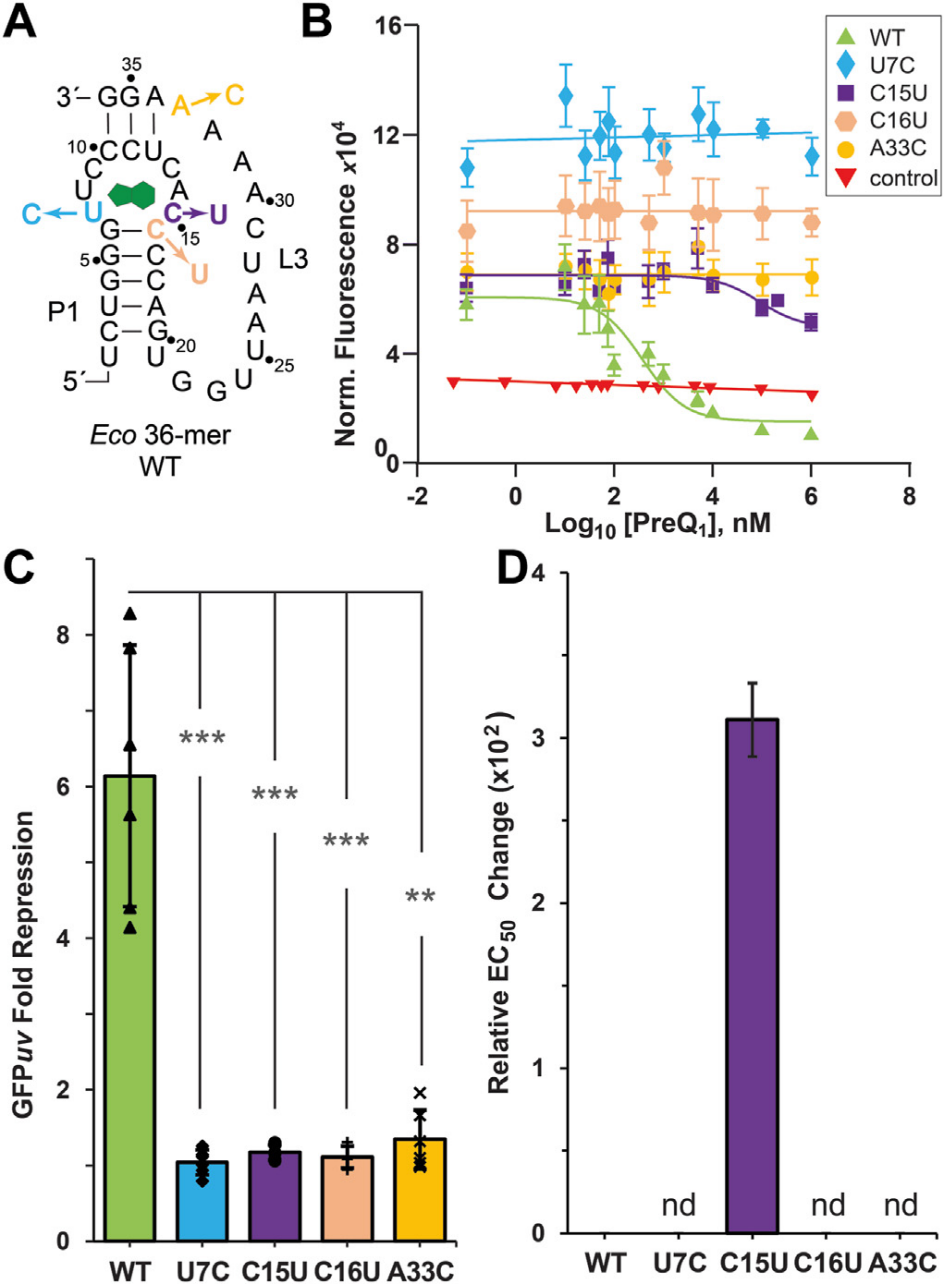
**Schematic diagram showing effect of mutations in WT *Eco* preQ**_**1**_**-I**_**III**_**riboswitch on switching in a bacterial reporter assay.***A*, secondary structure of the modified WT *Eco* 36-mer in a GFP*uv* reporter construct. Arrows indicate specific mutations to conserved bases. *B*, preQ_1_-dependent dose-response curves of reporter-gene GFP*uv* normalized fluorescence emission comparing the WT *Eco* riboswitch with various mutants. *E. coli* Δ*queC* cells containing the riboswitch reporter gene were grown with varying amounts of preQ_1_ and fluorescence was measured for each concentration. Curve fits are based on the average of six biological measurements. The negative control lacked the riboswitch and had no functional SDS. *C*, bar plot showing fold repression of GFP*uv* fluorescence for WT and mutants; errors are SEM for six biological replicates; ∗∗ *p* < 0.01 and ∗∗∗ *p* < 0.001. *D*, the EC_50_ fold change of each mutation relative to WT; nd: the mutant EC_50_ could not be determined. Data from *B*–*D* are summarized in Table 4.

To validate the switching activity of the type III *Eco* riboswitch (Fig. 8*A*), we placed the aptamer upstream of a GFP*uv* reporter gene to assess its function in live *E. coli*. The WT *Eco* riboswitch produced a preQ_1_-dependent dose response in which GFP*uv* emission decreased in the presence of the metabolite (Fig. 8*B*). The WT EC_50_ was 291.4 ± 1.6 nM with a 6.1-fold overall repression by preQ_1_ (Fig. 8*C* and Table 4). The *Eco* riboswitch gene-regulatory capability is comparable to the *Can* type I preQ_1_-I riboswitch in terms of its EC_50,1_ (96 ± 14 nM), but the *Eco* riboswitch had a 2.4-fold reduction in overall gene repression (21). Notably, the *Can* riboswitch senses two preQ_1_ equivalents in a single binding pocket (Fig. 1*D*) and produces its highest repression at preQ_1_ levels over the EC_50,2_ of 7.1 ± 0.4 μM (21). Compared to other known riboswitches, the overall level of repression by the *Eco* riboswitch is considered to be ‘strong’ (40).

**Table 4:** Reporter gene activity for *Eco* WT and mutant riboswitches

*GFPuv Construct*	*EC*_*50*_*, nM*	*EC*_*50 (re)l*_^a^	*Fold Repression*
*Eco WT*	291.4 ± 1.6	–	6.1 ± 1.7
*Eco U7C*	–	–	1.0 ± 0.2
*Eco C15U*	90,573 ± 3500	311	1.2 ± 0.1
*Eco C16U*	–	–	1.1 ± 0.1
*Eco A33C*	–	–	1.3 ± 0.4

a Calculated as *EC*_*50*, mutant_/*EC*_*50*, WT_.

We next analyzed how individual mutants affected regulation of GFP*uv* gene expression. Using the same three mutations described above, U7C and C16U (Fig. 8*A*) each showed significantly higher GFP*uv* fluorescence than WT but neither showed gene regulation, even at saturating levels of preQ_1_ (32) (Fig. 8*B* and Table 4). The higher fluorescence emission indicates that more GFP*uv* protein is produced in the presence of these mutations compared to WT. This observation is consistent with our structure wherein U7C is predicted to weaken preQ_1_ binding by disrupting only a single hydrogen bond (*i.e.*, U6 in Fig. 3*E*), whereas C16U disrupts the P1 stem in the pocket floor (*i.e.*, C15 in Fig. 3*C*). Notably, a previous study observed a similar effect for the preQ_1_-II riboswitch when the anti-SDS sequence was mutated to inhibit pairing with the SDS (39).

The A33C mutant (Fig. 8*A*) showed no gene regulation under saturating preQ_1_, as indicated by its flat dose response (Fig. 8*B*). Here, GFP*uv* fluorescence remained comparable to the WT gene-on state across all tested preQ_1_ doses. In the SPR analysis, we replaced A33 with a non-natural purine nucleobase, but for *in vivo* assays, we made the A33C mutation since cytosine preserves the SDS consensus 5'-(A/C)(A/U)GGA(A/G)AA (38) and can still hydrogen bond with the Watson-Crick face of adenine. Nonetheless, A33C likely disrupts two hydrogen bonds to preQ_1_ (*i.e.*, A27 in Fig. 5*D*) and ablates Hoogsteen edge interactions with U8 (*i.e.*, U7 in Fig. 5*D*).

Interestingly, mutation of the specificity base, C15, to uracil was the only mutant that showed a dose response (Fig. 8, *A* and *B*). The C15 mutant had an estimated EC_50_ of 90.6 ± 0.4 μM, which is approximately 311-fold weaker than WT (Table 4 and Fig. 8*D*). Full repression was not achieved even at saturating preQ_1_ levels of 1 mM, and thus the EC_50_ value could be considerably higher. As with the other mutants, the fold-repression was extremely weak (Table 4 and Fig. 8*C*). C-to-U mutations in the specificity bases of the type I *Can* riboswitch also increased EC_50_ values, but only by ∼60- (C17U) and ∼210-fold (C31U) over WT, corresponding to 6-fold and 2-fold repression (26). Our *Eco* riboswitch structure suggests that the C15U mutation not only disrupts interactions with preQ_1_ (*i.e.*, C14 in Fig. 3*E*) but also promotes a new cross-strand interaction with A33 (*i.e.*, A27 in Fig. 3*E*) that competes with preQ_1_ binding. This possibility is consistent with the substantially weakened ligand-binding properties of this mutant measured by SPR (Fig. 7*E*).

## Discussion

Here, we present the structure of the *Eco* riboswitch in complex with preQ_1_, the first cocrystal structure of a preQ_1_-I_III_ (class I type III) riboswitch bound to preQ_1_. The structure and function analysis is the first major characterization of a type III preQ_1_ riboswitch since type III (preQ_1_-I_III_) was first described as a distinct class I subgroup (9). Accordingly, all known preQ_1_ riboswitch classes and subtypes have now been characterized structurally. All three preQ_1_-I riboswitch subtypes fold as H-type pseudoknots that share multiple architectural features based on comparisons of representative structures corresponding to the *Can, Tte*, and *Eco* bacterial sequences (Fig. 4). Each riboswitch type adopts a highly compact fold that features a small, 5 to 6 base pair P1 stem (Fig. 3, *A* and *B*). The P1 helix is fortified in the minor groove by multiple A-amino kissing interactions from the A-rich L3 loop (Fig. S2*B*) that culminate in a quintuple-base transition motif that forms the binding pocket floor of the riboswitch (*e.g.*, Fig. 3*C*). Perhaps most remarkably, all three riboswitch types use common nucleobase determinants to recognize at least one equivalent of preQ_1_ in a homologous-binding pocket designated as the α site (Fig. 4*D*) (13, 14, 15, 21, 33). The structure reported here was determined by Mn^2+^ SAD phasing and reveals a site-bound ion at conserved nucleotide G4 (site I) that appears to stabilize the binding pocket floor to facilitate preQ_1_ sensing (Fig. 3*D*). An equivalent Mn^2+^ site was observed in the *Tte* preQ_1_-I_II_ riboswitch (21). The propensity of Mn^2+^ to coordinate at Mg^2+^ sites suggests an important role for divalent ion binding at equivalent positions in members of the preQ_1_-I riboswitch cohort.

Despite similarities with other riboswitches, the type III *Eco* riboswitch lacks several key interactions found in type I and II class I preQ_1_ riboswitches, suggesting that it may function as a ‘bare bones’ aptamer. This moniker is supported by the lower tolerance of the *Eco* riboswitch to mutations than representative type I *Can* and type II *Tte* riboswitches. As noted for preQ_1_ binding, U6 and A27 (crystal structure numbering) recognize the minor groove-edge equivalent of the metabolite but each position also engages in noncanonical interactions (Figs. 3*E* and 5*D*). In the *Eco* riboswitch, U6 pairs with the Hoogsteen edge of A26 in the binding pocket floor in a manner similar to U6 and A29 in the *Can* riboswitch, while the Hoogsteen edge of *Eco* A27 pairs with the Watson-Crick face of U6, similar to U7 and A30 in the *Can* riboswitch. However, the *Eco* riboswitch has poorer stacking upon the metabolite, wherein U11 sits above preQ_1_ and makes a canonical pair to A28, as well as a sugar-edge contact with A13 (Fig. 5*A*). By contrast, the type II *Tte* and type I *Can* riboswitch ceilings each stack a purine upon preQ_1_, which is held in place by base quadruple or triple interactions (Fig. 5, *B* and *C*). Moreover, the *Can* riboswitch evolved to bind two stacked metabolites, adding another tier of interactions that are absent in type II or III aptamers (Figs. 1, *C* and *D* and 3*B*). Hence, the dual metabolite recognition and RNA-folding roles of nucleobases U6 and A27 in the *Eco* riboswitch appear to render them less tolerant of mutations, as observed by the substantial loss of affinity for the U6C and A27Pur mutations (Fig. 7, *C* and *D*). Similarly, the equivalent U7C and A33C mutants were each unresponsive to preQ_1_ in bacterial reporter-gene assays and had unregulated GFP*uv* expression, even in the presence of saturating amounts of preQ_1_ (Fig. 8*B*).

Although the mode of preQ_1_-I_II_ riboswitch gene regulation is widely assumed to be representative of the entire class (13, 17, 18, 21, 33), our structure indicates that each class I subtype likely exhibits significant variability in its expression platform, particularly in the ceiling of the ligand-binding pocket and pseudoknot helix P2. Indeed, different P2 helix interactions bury the 5′-end of the SDS in response to preQ_1_ by the *Eco*, *Tte*, and *Can* riboswitches (Fig. 5, *D*–*F*). Some riboswitches, such as the cobalamin riboswitch (41), SAM-III riboswitch (42), NAD^+^-II riboswitch (43), and preQ_1_-II riboswitch (39), use the SDS to promote binding pocket formation; others, such as the preQ_1_-III riboswitch (30), the THF riboswitch (44), the TPP riboswitch (45), and SAM-V riboswitch (46), place the SDS distal to the metabolite-binding pocket. The preQ_1_-I_I_ and preQ_I_-I_II_ riboswitches belong to the latter category wherein the SDS does not directly form the metabolite pocket. Instead, these type I and II preQ_1_ riboswitches bury the first two SDS nucleotides above the pocket ceiling approximately 6 to 10 Å from the ligand (13, 14, 21, 26, 47) (Fig. 5, *E* and *F*). The mode of 5′-SDS sequestration differs between the two folds, but each has the first position of the SDS, an adenine, engaged in a noncanonical interaction and the second position, a guanine, engaged in a canonical Watson-Crick base pair (13, 14, 21, 26, 47) (Fig. 5, *E* and *F*). However, the *Eco* preQ_1_-I_III_ riboswitch integrates the SDS directly into the binding pocket, where the 5′-most adenine contacts the preQ_1_ metabolite *via* two hydrogen bonds (Fig. 3*E*). Thereafter, the SDS extends into the ceiling to participate in a base quartet (Fig. 5*A*) and is sequestered strongly by canonical Watson-Crick interactions that compose the remainder of P2 (Fig. 5*D*). Direct metabolite sensing by the SDS is rare but has been observed in the SAM/SAH riboswitch (48, 49) and the NAD^+^-II riboswitch (43) wherein the SDS interacts directly with the metabolite through one or more hydrogen bonds. Relatively speaking, the type III *Eco* riboswitch is somewhat distinct because the Watson-Crick face of the 5′-SDS adenine (A27) directly reads the preQ_1_ edge to confer specificity and affinity (Fig. 3*E*).

When this study was initiated, there was some ambiguity concerning type III riboswitch specificity, in part because the preQ_1_-I_III_ riboswitch regulates expression of the *yhhQ* gene, which encodes a transporter that salvages both preQ_1_ and preQ_0_ with an apparent preference for preQ_0_ (28). Moreover, the type II *Tte* riboswitch showed only a 17-fold preference for preQ_1_ over preQ_0_ (13). These factors suggested that the type III *Eco* riboswitch might prefer preQ_0_ binding over preQ_1_, as suggested in a previous in-line probing analysis (9). Our analyses show that, even though the binding pockets of *Tte* and the *Eco* riboswitch are nearly identical, the *Eco* riboswitch has a greater reduction in affinity for preQ_0_ than preQ_1_ (∼138-fold for *Eco* reduction *versus* 17-fold for *Tte*; Fig. 4*D*) (13, 14, 21). The greater basis for the discrimination between preQ_1_ and preQ_0_ by the *Eco* riboswitch appears to be the electronegative character of the nitrile group on the 7-position of preQ_0_, which is unprotonated (Fig. 6*B*). In the context of the *Eco*-binding pocket, the preQ_0_ nitrile group faces the O6 group of G5 and the negatively charged backbone at C12 (Fig. 6*D*), which are receptive to hydrogen bond donors but not to hydrogen bond acceptors. The noncomplementary charge between the nitrile group and the oxygen-rich RNA environment provides a plausible basis for the 20-fold increase in *k*_off_ of preQ_0_ compared to preQ_1_ (Table 3). Notably, the *Tte* riboswitch discriminates much less against preQ_0_ (*i.e.*, a 17-fold difference in *K*_D_) and exhibits no equivalent phosphate backbone interaction with the preQ_1_ methylamine. Accordingly, the nonbridging oxygen of C12 in the *Eco* riboswitch likely serves as a major specificity determinant. These results lead us to hypothesize that the *yhhQ* transport gene may have a preQ_1_ import preference in a cellular context.

We next considered the evolution of the α-site preQ_1_ pocket shared by type I, II, and III riboswitches, as revealed by this investigation (Fig. 4*D*). The preQ_1_-I_I_ riboswitch shows the highest number of riboswitch sequences per nucleotide (1) with >1500 representatives (Fig. 1*B*), as well as greater phylogenetic diversity than all other preQ_1_-I subtypes (9). Type I riboswitches also show many more representatives than type II in the *Clostridia* class (9), which is one of the most ancient groups of bacteria (50). By contrast, type III riboswitches are found almost exclusively in proteobacteria (9). Given the prevalence and ancient origins of type I riboswitches, as well as its conservation of the α-type preQ_1_-binding site (26), we speculate that the preQ_1_-I_I_ riboswitch was the parent of the other two preQ_1_ riboswitch subtypes. Loss of preQ_1_ recognition at the β-type preQ_1_-binding site of type I riboswitches likely occurred through multiple point mutations that formed a new ceiling while preserving α-site binding to maintain bacterial fitness. Differences in the pocket ceiling in the type II and type III riboswitches (Fig. 5, *B* and *C*) suggest that the β-binding site was lost more than once during bacterial evolution.

Finally, even though covariation data (9) correctly predicted that the three preQ_1_-I riboswitch types adopt the same fold (Fig. 4*A*), the structural data reveal that, despite their common α-site preQ_1_-binding pockets, these three preQ_1_ riboswitches have three different modes of SDS sequestration (Fig. 5, *D*–*F*). Accordingly, our results demonstrate that riboswitch subgroups within the same class can produce substantially different modes of gene regulation and metabolite recognition (21, 47). We predict that additional bacterial genomic sequences will reveal new riboswitch subclasses for existing classes that will likely have novel modes of metabolite recognition and new ways to control expression platform accessibility.

## Experimental procedures

### RNA synthesis and purification

All RNA was synthesized by Horizon Discovery, Inc. Except for biotinylated RNA, each sequence was deprotected according to the manufacturer’s instructions with heating adjusted to 65 °C for 30 min. RNA was purified using denaturing PAGE with 15% gels, followed by DEAE chromatography as described (51) with use of DEAE buffer comprising 0.02 M Na–Hepes pH 6.8, 0.1 M ammonium acetate, and 0.002 M EDTA. Rapid UV shadowing was used to prevent sample damage (52). RNA was desalted on PD-10 columns (Cytiva Corp), lyophilized, and stored at −20 °C.

### Isothermal titration calorimetry

Lyophilized RNA was prepared for ITC experiments as described (26). A typical ITC experiment used a PEAQ-ITC instrument (Malvern Panalytical Ltd., UK) with 10 μM RNA in the cell and 100 μM preQ_1_ (Lead Gen Labs, LLC) in the syringe. A total of 19 injections were made with a 0.5 μl technical injection and a 4 μl volume was used for all subsequent injections that were spaced 150 s apart. Best fits of thermograms were determined using a single-sites–binding model with the MicroCal analysis software for the PEAQ-ITC (Malvern Panalytical Ltd., UK). All experiments were performed in duplicate. Thermodynamic parameters are listed in Table 1. Sequences used in ITC are provided in Table S1 of the Supporting Information.

### Crystallization and flash freezing

Lyophilized *Eco* 30-mer RNA was dissolved in 20 μl 0.01 M Na-cacodylate pH 7.0. The RNA concentration was adjusted to 0.5 mM and the solution was heated at 65 °C for 3 min in an aluminum heat block. A folding mix comprising 0.01 M Na-cacodylate pH 7.0, 0.004 M MgCl_2_, and 0.1 M preQ_1_ was also heated at 65 °C for 3 min before an equal volume was added slowly to the RNA with gentle mixing. The RNA-folding mix solution was heated for an additional 3 min and slowly cooled to room temperature in the aluminum block.

Crystals were grown in VDX Plates (Hampton Research) using 2 μl of folded RNA at 250 μM combined with 1 μl well solution comprising 0.005 M MnCl_2_ tetrahydrate, 31% MPD, 0.05 M Na-cacodylate pH 6.0, 0.012 spermine–HCl, 0.1 M KCl, and 0.002 M MgCl_2_. Crystals grew in ≥2 weeks at 20 °C and were harvested using nylon loops attached to 18 mM copper pins. The crystals were serially transferred for 3 min into synthetic mother liquors containing 35% and 40% MPD for cryoprotection and then flash-frozen by plunging into N_2_ (*l*). Crystals were shipped to the Stanford Synchrotron Radiation Lightsource for data collection.

### X-ray diffraction and structure determination

X-ray diffraction data at 100 K were collected remotely on beamline 12-2 using Blu-Ice (53) software and the Stanford automounter (54) at a wavelength of 1.85 Å with a Δφ of 0.15º and a transmission setting of 10%. Data were recorded from three unique volumes of a plate-shaped crystal using an EIGER X 16M detector (Dectris). A total of 8491 frames were used for structure determination with 2,399, 897, and 5195 frames from the first, second, and third volumes. Data collection strategies were generated in Web-Ice (55), and the diffraction data were reduced with the autoxds script (https://smb.slac.stanford.edu/facilities/software/xds/#autoxds_script), which uses XDS and the CCP4 programs POINTLESS, AIMLESS, and TRUNCATE (56, 57). The x-ray intensity and data reduction statistics are presented in Table 2.

Phasing efforts using molecular replacement were unsuccessful based on search models derived from preQ_1_-I_I_ and preQ_1_-I_II_ riboswitches (15, 21, 26). Accordingly, the phase problem was determined by SAD based on Mn^2+^ ions cocrystallized with the riboswitch. A significant anomalous diffraction signal was detected to 2.57 Å resolution in AIMLESS based on the point at which the anomalous correlation coefficient (CC_anom_) between half datasets dropped below a threshold of 0.15. Phenix.HySS (58) identified four Mn^2+^ ions in the substructure, which produced an initial figure of merit of 0.30 (Table 2). The Phenix.Autosol (58) noise-filtered map resulted in a figure of merit of 0.62 for the full-resolution range. Phenix.Autobuild (58) produced an initial model comprising 21 polyA nucleotides and 17 waters, yielding a map-model CC of 0.38. Calculation of the Matthews’ coefficient and solvent content (59) produced values of 2.52 Å^3^ Da^-1^ and 67%, suggesting that two 30-mer molecules were present per asymmetric unit. The backbone of the initial A-chain model was rebuilt manually into solvent-flipped SAD-phased electron density maps using COOT (60), yielding a nearly complete chain of the riboswitch. This model was then used as a search model in molecular replacement to place the second copy, which was more poorly defined in initial maps than the first copy, into the asymmetric unit. The resulting quality control metrics for molecular replacement included a translation function Z-score of 15.7 and a log-likelihood gain of 587. The model was built iteratively using COOT. Model refinement was done with Phenix.Refine (58) with TLS groups added as described (61). Refinement statistics are provided (Table 2). The anomalous scattering corrections for Mn^2+^ were refined using Phenix.Refine (58). Least-squares superpositions of paired atoms were performed in LSQKAB as implemented in CCP4 (57, 62). In Figure 4, chain A of the *Eco* (type III) riboswitch was superimposed on chain A of the bound-state *Can* (type I) riboswitch (PDB code 7REX) (26) from positions 2 to 7 (2–7) and 13 to 30 (16–33), where parenthetical values correspond to the *Can* riboswitch. Chain A of the *Eco* riboswitch was superimposed onto chain A of the bound-state *Tte* (Type II) riboswitch (PDB code 6VUI) (21) from positions 2 to 11 (2–11), 13 to 20 (14–21), and 23 to 30 (25–32), where parenthetical values correspond to the *Tte* riboswitch. All structure representations were made with PyMOL (Schrödinger, Inc).

### Surface plasmon resonance

NeutrAvidin-conjugated CM5 chips were prepared on a Biacore T200 instrument (Biacore Inc) using reagents from the manufacturer. Riboswitch samples with 5′-biotinylation were obtained in deprotected and desalted form (Horizon Discovery) (Table S2 of the Supporting Information). Biotinylated WT and mutant *Eco* riboswitches were dissolved in 200 μl 0.01 M Na-cacodylate pH 7.0. The RNA was folded by heating at 90 °C for 3 min and cooled quickly on ice for 3 min, during which an equal volume of ice-cold folding mix (0.01 M Na-cacodylate pH 7.0 and 0.006 M MgCl_2_) was added slowly. The RNA was then warmed to room temperature. The concentration of folded RNA was adjusted to 0.5 mM in SPR running buffer (0.01 M Na–HEPES pH 7.5, 0.1 M NaCl, and 0.003 M MgCl_2_). Each folded riboswitch was injected over NeutrAvidin-conjugated flow cells to give a surface density of ∼ 3000 response units; the reference flow cell had only NeutrAvidin.

For kinetic experiments, the association phase analysis comprised 120 s injections of preQ_1_ or preQ_0_ conducted at concentrations ranging from 78 nM to 2500 nM for preQ_1_ and from 1.95 μM to 62.5 μM for preQ_0_ at 100 μl min^-1^. PreQ_1_ solutions were prepared by diluting a preQ_1_ stock in water with SPR running buffer. PreQ_0_ solutions were prepared by dissolving enzymatically prepared preQ_0_ (63) in SPR running buffer followed by heating as described (14). Dissociation was monitored for 300 s. Regeneration was conducted by injection of 3 M guanidine hydrochloride solution for 45 s followed by a 120 s incubation in running buffer flowing at 30 μl min^-1^.

Equilibrium binding experiments involved injections of preQ_1_ lasting 120 s at 30 μl min^-1^ and were conducted with concentrations ranging from 1.25 to 200 μM for G6DAP-C16U and 9.7 to 10,000 μM for C15U, U7C, and A33Pur mutants. For these variants, a 400 s dissociation phase in running buffer was used. Regeneration was accomplished as described above.

All measurements were made in duplicate at 25 °C with a data collection rate of 10 Hz. Experimental data were processed using Biacore T200 Evaluation software version 3.2.0.5 (GE Healthcare). The double referencing method was used to remove instrumental and bulk shift effects (64). Buffer-subtracted sensorgrams for the kinetic binding data were then fit to a 1:1 binding interaction model to determine the rate constants (*k*_on_ and *k*_off_) and the apparent equilibrium binding constant (*K*_D_). To evaluate the steady-state equilibrium binding data, the response during the equilibrium binding phase (R_eq_) was calculated by averaging the response over 5 s just before the end of the metabolite injection. Equilibrium binding data were fit using nonlinear least-squares curve fitting in Prism version 9 (GraphPad, Inc) to a one-site binding equation to determine *K*_D_. Kinetic and equilibrium binding constants are provided in Table 3.

### In-cell GFP*uv* reporter assays

The WT *Eco* riboswitch was inserted into the pBR327-*Lrh*(WT)-GFP*uv* plasmid upstream of the GFP*uv* reporter gene as described (32). Riboswitch variants were prepared by site-directed mutagenesis (GenScript Inc) based on the WT sequence, except that the closing base pair in P1 was changed from U1 to C and A19 to G to avoid undesired folding of the GFP*uv* transcript. All constructs were verified by DNA sequencing. Sequences are provided in Table S3 of the Supporting Information.

The reporter assay was performed as described (32, 39) with some exceptions. *E. coli* strain JW0434-1 Δ*queC* cells (Horizon Discovery), which cannot synthesize preQ_0_, were transformed with the desired plasmid and grown on chemically-defined salt broth (CSB) agar plates containing ampicillin (100 *μ*g mL^−1^) and kanamycin (50 *μ*g mL^−1^). Single colonies were isolated to inoculate overnight liquid cultures of 3 ml CSB-*amp-kan* media. These cultures were used to inoculate 2 ml of fresh CSB-*amp-kan* liquid media containing varying concentrations of preQ_1_: 0, 0.1 nM, 10 nM, 25 nM, 50 nM, 75 nM, 100 nM, 500 nM, 1 *μ*M, 5 *μ*M, 10 *μ*M, 100 *μ*M, and 1 mM. A negative control was also evaluated at each preQ_1_ concentration wherein the WT SDS was replaced with the complementary sequence.

Six biological replicates were measured for each concentration. All measurements and analyses were performed as described (32) using Prism (GraphPad Software, Inc); replicates of each construct were compared using the “compare datasets” function before analysis. Additionally, a negative control was evaluated at each preQ_1_ concentration (32). The WT *Eco* riboswitch and C15U curves showed single transitions described by 3 parameter fits of log(inhibitor) dose *versus* response.

The *p* value for WT *Eco versus* U7C was 0.0001 (*t* = 7.2106, df = 10, 95% confidence interval = 3.523–6.674). The *p* value for WT *Eco versus* C15U was 0.0009 (*t* = 7.0469, df = 5, 95% confidence interval = 3.156–6.781). The *p* value for WT *Eco versus* C16U was 0.0008 (*t* = 7.1183, df = 5, 95% confidence interval = 3.212–6.844). The *p* value for WT *Eco versus* A33C was 0.0012 (*t* = 6.6468, df = 5, 95% confidence interval = 2.942–6.653).

## Data availability

All data produced in this investigation are provided in the manuscript. Any additional information or reagents can be obtained by contacting the corresponding author. Coordinates and structure factors of the refined *Eco* preQ_1_-IIII riboswitch structure were deposited into the Protein Data Bank under accession code 8fza.

## Supporting Information

This article contains supporting information.

## Conflict of interest

The authors declare that they have no conflicts of interest with the contents of this article.
