# Supplementary material for: Structure and function analysis of a type III preQ1-I riboswitch from Escherichia coli reveals direct metabolite sensing by the Shine-Dalgarno sequence
Source: J Biol Chem. 2023 Sep 1;299(10):105208. doi: 10.1016/j.jbc.2023.105208 (PMC10622847; doi:10.1016/j.jbc.2023.105208)
Supplement: Supporting information [file mmc1.pdf]

Supporting Information

Structure and function analysis of a type III $preQ_1$-I riboswitch from *Escherichia coli* reveals direct metabolite-sensing by the Shine-Dalgarno sequence

Griffin M. Schroeder[1,2,3], Daniil Kiliushik[1,2], Jermaine L. Jenkins[1,2], and Joseph E. Wedekind[1,2,*]

[1] Department of Biochemistry and Biophysics, University of Rochester School of Medicine and Dentistry, Rochester, NY, 14642, USA

[2] Center for RNA Biology, University of Rochester School of Medicine and Dentistry, Rochester, NY, 14642, USA

* To whom correspondence should be addressed. Tel: +1 585-273-4516; Fax: +1 585 275-6007; Email: joseph.wedekind@rochester.edu

[3] Present Address: Griffin M. Schroeder, Arrakis Therapeutics, Waltham, MA, 02451, USA

ORCIDs: 0000-0001-6354-752X (G.M.S), 0000-0003-3055-5122 (D.K), X0000-0003-2548-3275 (J.L.J.), 0000-0002-4269-4229 (J.E.W)

Supporting Figures

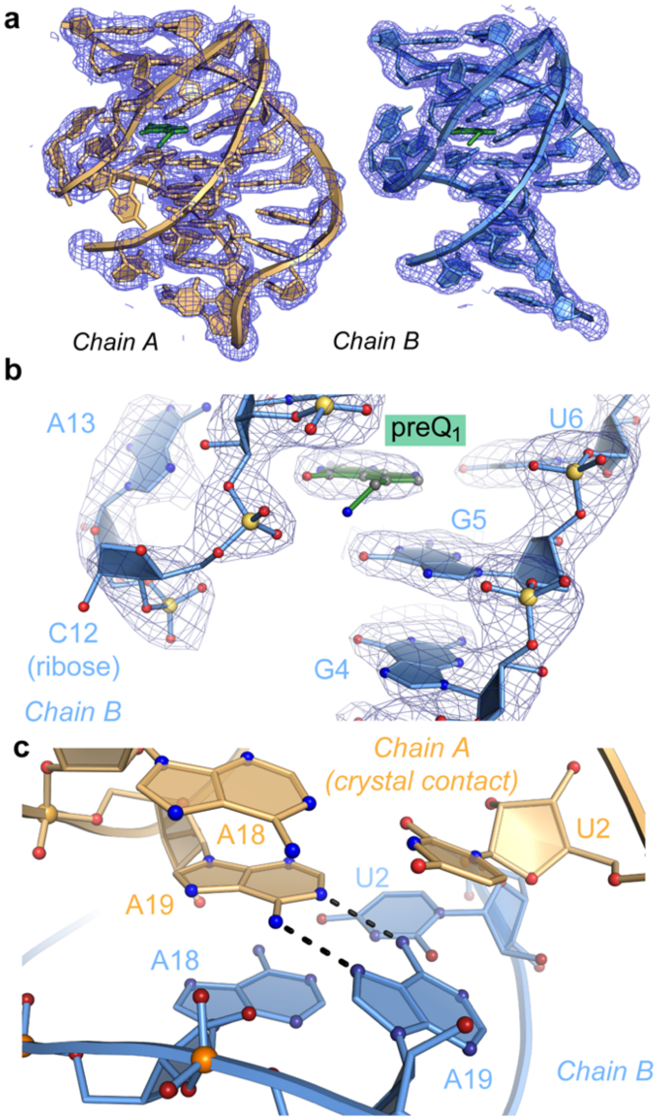

Figure S1: Structural details of the *E. coli* preQ$_1$-I$_{III}$ riboswitch. (**a**) Final 2mF_o-DF_c density maps contoured to 1.1σ around each riboswitch chain in the asymmetric unit. Chain A is colored in orange and is completely bathed in electron density. Chain B is colored in blue and shows a break in electron density in loop L3. PreQ$_1$ is colored in green in each chain. Ribbon diagrams of the final refined models are shown. (**b**) Close up view of C12 with 2mF_o-DF_c density contoured at 1.1σ. Only the phosphate and ribose of C12 are modelled in chain B. (**c**) A crystal contact occurs between the Watson-Crick face of A19 in chain A and the Hoogsteen edge of A19 in chain B. Position U1 is disordered in each chain.

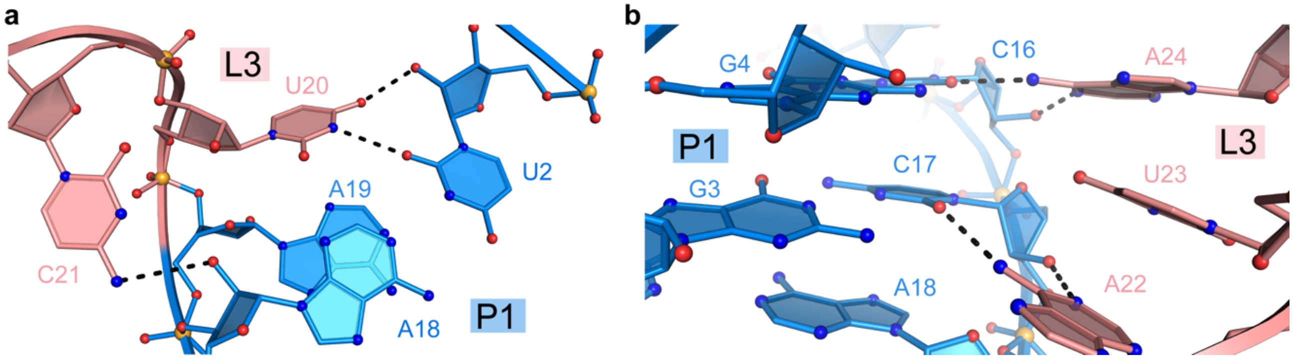

Figure S2: Interactions between the minor-groove P1 helix and the L3 linker. (**a**) Ball-and-stick diagrams showing hydrogen bonds between U20 and C21 of loop L3 to P1 minor-groove bases U2 and A18. This region was modified to promote crystal formation. (**b**) A-amino kissing interactions between A22 and A24 of L3 to the P1 minor groove at C17 and C16.

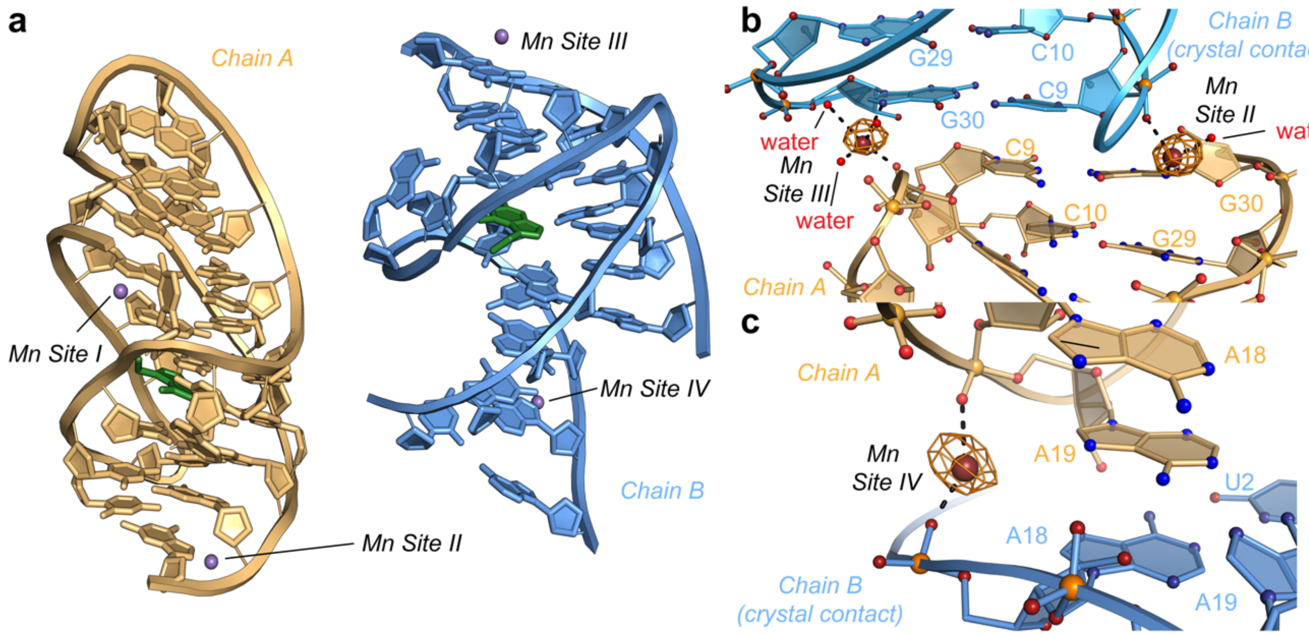

Figure S3: Ribbon diagrams showing manganese ions. (**a**) Ribbon diagrams depicting global view of chains A and B in the asymmetric unit. Manganese binding sites are labelled I-IV. (**b**) Mn^{2+} ions at sites II & III mediate crystal contacts at the C9-G30 base pair of helix P2, which forms a flush stack at the interface between chains. The anomalous difference Fourier map is contoured at 8.5σ. (**c**) Mn^{2+} at site IV mediates a crystal contact between non-bridging phosphate oxygens of A19 in chain A and A18 in chain B. The anomalous difference Fourier map is contoured at 6.0σ.

Table S1: Riboswitch Sequences Used for ITC

Name	RNA Sequence
WT *Eco* riboswitch 36-mer	AUUGGGUUCCCUCACCCCAAUGGUUAAUCAAAAAGG
35-mer	AUUGGGUUCCCUCACCCCAAUGUUAAUCAAAAAGG
34-mer	AUUGGGUUCCCUCACCCCAAUUUAAUCAAAAAGG
33-mer	AUUGGGUUCCCUCACCCCAAUUAAUCAAAAAGG
30-mer	UUGGGUUCCCUCACCCCAAUCAUAAAAAGG

Table S2: Riboswitch Sequences Used for SPR

Name	RNA Sequence
WT *Eco* riboswitch	Bi-AUUGGGUUCCCUCACCCCAAUGGUUAAUCAAAAAGG
G6DAP/C16U	Bi-AUUGG(DAP)UUCCCUCACUCCAAUGGUUAAUCAAAAAGG
U7C	Bi-AUUGGGCUCCCUCACCCCAAUGGUUAAUCAAAAAGG
C15U	Bi-AUUGGGUUCCCUCAUCCCAAUGGUUAAUCAAAAAGG
A33Pur	Bi-AUUGGGUUCCCUCACCCCAAUGGUUAAUCAAA(Pu)AAG

Table S3: Riboswitch Sequences Used for GFP*uv* Reporter Assays

Name	DNA Sequence in plasmid pBR327[a]
WT *Eco* riboswitch	ATGCATAAGGCTCGTATAATTGAGTT**A**TTAAATT*CTGGGTTCCCTCACCCCAGTGGTTAATCAAA* ***AAGG*****AGG**ATTCACACATGCTGATGATTACGCCAAGCTT[b]
U7C	*ATGCAT*AAGGCTCGTATAATTGAGTT**A**TTAAATT*CTGGGCTCCCTCACCCCAGTGGTTAATCAAA* ***AAGG*****AGG**ATTCACACATGCTGATGATTACGCCAAGCTT
C15U	ATGCATAAGGCTCGTATAATTGAGTT**A**TTAAATT*CTGGGTTCCCTCATCCCAGTGGTTAATCAAA* ***AAGG*****AGG**ATTCACACATGCTGATGATTACGCCAAGCTT
U16C	ATGCATAAGGCTCGTATAATTGAGTT**A**TTAAATT*CTGGGTTCCCTCACTCCAGTGGTTAATCAAA* ***AAGG*****AGG**ATTCACACATGCTGATGATTACGCCAAGCTT
A33C	ATGCATAAGGCTCGTATAATTGAGTT**A**TTAAATT*CTGGGTTCCCTCACCCCAGTGGTTAATCAAA* ***CAGG*****AGG**ATTCACACATGCTGATGATTACGCCAAGCTT

[a] Sequences are flanked by unique *NsiI* and *Hind*III restriction sites.

[b] The underlined, bold A is the transcription start site. The italicized sequence is the *Eco* riboswitch. The bold sequence is the Shine-Dalgarno sequence. Underlined ATG is the start codon.
